# Population Structure Limits Parallel Evolution in Sticklebacks

**DOI:** 10.1093/molbev/msab144

**Published:** 2021-05-06

**Authors:** Bohao Fang, Petri Kemppainen, Paolo Momigliano, Juha Merilä

**Affiliations:** 1 Ecological Genetics Research Unit, Organismal and Evolutionary Biology Research Programme, Faculty of Biological and Environmental Sciences, University of Helsinki, Helsinki, Finland; 2 Research Division for Ecology and Biodiversity, The School of Biological Sciences, The University of Hong Kong, Hong Kong SAR, China

**Keywords:** adaptation, genetic diversity, isolation by distance, population differentiation, parallel evolution, stickleback

## Abstract

Population genetic theory predicts that small effective population sizes (*N*_e_) and restricted gene flow limit the potential for local adaptation. In particular, the probability of evolving similar phenotypes based on shared genetic mechanisms (i.e., parallel evolution), is expected to be reduced. We tested these predictions in a comparative genomic study of two ecologically similar and geographically codistributed stickleback species (viz. *Gasterosteus aculeatus* and *Pungitius pungitius*). We found that *P. pungitius* harbors less genetic diversity and exhibits higher levels of genetic differentiation and isolation-by-distance than *G. aculeatus*. Conversely, *G. aculeatus* exhibits a stronger degree of genetic parallelism across freshwater populations than *P. pungitius*: 2,996 versus 379 single nucleotide polymorphisms located within 26 versus 9 genomic regions show evidence of selection in multiple freshwater populations of *G. aculeatus* and *P. pungitius*, respectively. Most regions involved in parallel evolution in *G. aculeatus* showed increased levels of divergence, suggestive of selection on ancient haplotypes. In contrast, haplotypes involved in freshwater adaptation in *P. pungitius* were younger. In accordance with theory, the results suggest that connectivity and genetic drift play crucial roles in determining the levels and geographic distribution of standing genetic variation, providing evidence that population subdivision limits local adaptation and therefore also the likelihood of parallel evolution.

## Introduction

Parallel evolution—defined here as the evolution of similar phenotypes in multiple independently colonized populations via selection on alleles that are identical by descent—is considered to be strong evidence for the role of natural selection in evolutionary change (Schluter et al. 2004). Adaptation from standing genetic variation (SGV) is thought to be the dominant route to parallel evolution among recently diverged populations (Conte et al. 2012; Ord and Summers 2015). Because genetic drift erodes SGV and barriers to gene flow can prevent beneficial alleles from reaching populations adapting to specific local habitats (Lenormand 2002; Barrett and Schluter 2008; Feulner et al. 2013), both the effective population size (*N*_e_) of the ancestral population (MacPherson and Nuismer 2017; Thompson et al. 2019) and gene flow (Ralph and Coop 2015; Bailey et al. 2017; Lee and Coop 2017) are expected to play key roles in determining the probability of parallel evolution. Both factors are expected to affect the heterogeneity in the geographic distribution of SGV across the distribution ranges of species. However, despite increasing interest to understand the drivers of parallel evolution over the past decade (e.g., Arendt and Reznick 2008; Elmer and Meyer 2011; Conte et al. 2012; Stern 2013; Elmer et al. 2014; Rosenblum et al. 2014; Stuart et al. 2017; Bolnick et al. 2018; Barghi et al. 2019), little effort has been placed to investigate the role of geographic heterogeneity in SGV (but see Lee and Coop 2017; Fang et al. 2020; Kemppainen et al. 2021). One way to test whether heterogeneity in pools of SGV determines the probability of parallel evolutionary responses is to investigate genetic structure and local adaptation in pairs of codistributed and ecologically similar species that differ in their dispersal potential and population size, and hence, in the degree of heterogeneity of their pools of SGV.

The three-spined stickleback (*Gasterosteus aculeatus*) is an iconic model species used to study genetic parallelism. A multitude of studies has shown that the independent colonization of freshwater habitats across the global distribution range of this species has led to substantial, and often (but not always) parallel marine–freshwater-associated genetic differentiation (Colosimo et al. 2005; Hohenlohe et al. 2010; DeFaveri et al. 2011; DeFaveri et al. 2012; Jones et al. 2012; Hohenlohe and Magalhaes 2019; Fang et al. 2020). Despite the near circumpolar distribution of *Gasterosteus* sticklebacks, only three taxonomically valid species have been recognized in this genus (viz. *G. aculeatus*, *G. wheatlandi*, and *G. nipponicus*; Eschmeyer et al. 2017). In contrast, the circumpolarly distributed stickleback fishes in the genus *Pungitius* harbor at least eight taxonomically valid species (Takahashi et al. 2016; Eschmeyer et al. 2017; Guo et al. 2019), and there is evidence that the level of genetic differentiation among local populations in this genus greatly exceeds that seen among local populations of *Gasterosteus* (DeFaveri et al. 2012; Merilä 2013; Kemppainen et al. 2021; but see Raeymaekers et al. 2017). Thus, comparative genetic studies of these two codistributed species can provide an opportunity to gain novel insight into how differences in population structure, and thus in the distribution of SGV, may translate into differences in the probability of genetic parallelism. However, apart from two geographically restricted studies (DeFaveri et al. 2012; Raeymaekers et al. 2017), there has been no attempt to study and compare the levels of genetic variability and divergence among *Gasterosteus* and *Pungitius* taxa in a quantitative manner in a broad geographic context.

There is generally a greater abundance of three- than nine-spined sticklebacks in the sea (e.g., Quinn and Light 1989; Cowen et al. 1991; Jurvelius et al. 1996; Ojaveer et al. 2003) which is likely to limit gene flow and contribute to substantial genetic isolation by distance (IBD) in marine nine-spined sticklebacks. Consistent with this, earlier work suggests that the pool of SGV is indeed reduced and more fragmented in nine- compared with three-spined sticklebacks (DeFaveri et al. 2012; Kemppainen et al. 2021). To gain a holistic view of factors that influence such differences in SGV, using comprehensive geographic sampling and high-density population genomic data, we first re-assess the differences in both phylogenetic histories and population demographic parameters between these two species. We then formulate and test the hypothesis that due to a higher geographic heterogeneity in SGV in nine-spined sticklebacks, this species will show a lower prevalence of parallel evolution in response to freshwater colonization than the three-spined stickleback.

## New Comparative Outlier Analyses Approach

The degree of genetic parallelism in response to freshwater colonization in three- and nine-spined sticklebacks was assessed in two steps. First, Linkage Disequilibrium Network Analyses (LDna; Kemppainen et al. 2015; Li et al. 2018; Fang et al. 2020) was used to partition data into correlated sets of loci (LD clusters), followed by linear mixed models (LMMs) testing for associations between Principal component (PC)-coordinates based on loci from the LD clusters and ecotype (treated as a binary trait) while controlling for *P* value inflation due to relatedness and other confounding factors. For these analyses, only samples from the Atlantic region were used, and the data sets were normalized such that the same number of loci were analyzed for both species after accounting for differences in sequencing coverage and genetic diversity (see supplementary method 1, Supplementary Material online). Ultimately, 882,125 and 1,355,325 SNPs (in the form of genotype likelihoods) were used in the downstream comparative LDna analyses in three- and nine-spined sticklebacks, respectively.

### Complexity Reduction Using LDna Analyses

Because LDna relies on pairwise LD estimates among all loci, it is not feasible to consider all pairwise comparisons at once for large data sets. Instead, we adopted a nested approach, starting with LDna within windows within chromosomes (LDna-1, sensu Li et al. 2018; supplementary method 1, Supplementary Material online), followed by LDna within chromosomes (LDna-2) and finally LDna for genome-wide SNPs (LDna-3) as described in Fang et al. 2020, with some modifications (an overview of this approach is given in supplementary figure 1, Supplementary Material online, and further details are given in supplementary method 1, Supplementary Material online). First, LD clusters were defined by a single parameter, the minimum number of edges in the cluster (*|E|*_min_), rendering the previously used *λ*_lim_ parameter (that determines how different LD signals are between different clusters) obsolete.

Second, instead of using one SNP (rSNP) to represent a cluster in the subsequent LDna-step, the final LDna-3 was based on a pair-wise correlation matrix of *r*^2^ between PC1-coordinates extracted from the LDna-2 clusters entering LDna-3 analyses (supplementary method 1, Supplementary Material online). The covariance matrices were estimated from genotype likelihoods using PCAngsd (Meisner and Albrechtsen 2018) from all loci in a given LDna-2 cluster. The first PC typically explained >90% of the variation in each LD cluster and in supplementary figure 2, Supplementary Material online, we demonstrate that these coordinates can be regarded as “synthetic multi-locus alleles” (SMLAs). Third, all LDna-1 clusters with more than SNP_min_ number of loci and that were not part of any LDna-2 cluster were also included in the final LDna-3 analyses. Fourth, LDna-3 clusters were determined by the parameter Cor_th_, which specifies the weakest link (*r*^2^ value) in a cluster that is allowed between LDna-1 and LDna-2 clusters entering LDna-3. As such, decreasing parameter values for *|E|*_min_, SNP_min_, and Cor_th_ lead to many smaller clusters with few but highly correlated loci and *vice versa*.

There is a trade-off between (1) “under clustering” (i.e. analyzing many clusters that in reality reflect the same evolutionary phenomena leading to overly conservative corrections for multiple testing) and (2) “over clustering” (i.e., analyzing fewer and larger clusters but each with sets of less correlated loci). Although the latter leads to less conservative corrections for multiple testing (higher power), it likely also leads to weaker associations between the SMLAs and ecotype. Importantly, the parameter settings that maximizes the power to detect a particular genomic region of interest will depend on both the data set (numbers of loci and the underlying LD structure) and the genomic region in question. Here we solved this by testing a range of parameter settings for *|E|*_min_ [10, 20, 40], SNP_min_ [10, 20, 40], and Cor_th_ [0.8, 0.7, 0.6, 0.5] for both data sets. All LD values were estimated by *ngsLD* (Fox et al. 2019) based on genotype likelihoods. Further details are given in supplementary method 1, Supplementary Material online, and below.

### Linear Mixed Model Analysis (LMM) for Testing Associations between LD Clusters and Ecotype

We used LMM to test for associations between ecotype and the genetic variation explained by loci in LD clusters by regressing the SMLAs against ecotype treated as a binary trait [0, 1]. Using LMM to test associations between genotype and phenotype has previously been shown to be analogous to using permutation to test for allele frequency difference between two groups (Kemppainen et al. 2017), with the major benefit of LMM’s being computational speed and the ability to account for confounding factors such as relatedness. When not accounting for any potential confounding factors, the LMM-approach used here produces test statistics that are highly correlated with both *F*_ST_ and the cluster separation score (CSS; supplementary method 2 and fig. 2, Supplementary Material online), two commonly used metrics to detect genomic regions associated with parallel evolution in three-spined sticklebacks (Jones et al. 2012; Fang et al. 2020; Kingman et al. 2020). We used a modified version of the restricted maximum likelihood-based method EMMAX that allowed us to test for associations between SMLAs (rather than a single bi-allelic SNP at a time) from LD clusters and ecotype (supplementary method 3, Supplementary Material online).

Two approaches to control for multiple testing were used as follows: permutation (Li et al. 2018) (supplementary method 4, Supplementary Material online) and the Benjamini and Hochberg method also known as false discovery rate “FDR” (Benjamini and Hochberg 1995). In addition, two methods to control for *P* value inflation caused by relatedness were used: including a relatedness matrix ***A*** as a random effect (Kang et al. 2010) and genomic control (GC) (Price et al. 2010). Whenever FDR was used to control for multiplicity, we also iteratively estimated *P* value inflation as the linear slope *λ* between observed and expected (under the null-hypothesis) −log_10_(*P*) values before and after removing significant LD clusters from the data (the medians from all orthogonal combinations of *|E|*_min_, SNP_min_, and Cor_th_ were used). The reason for this was the exceptionally high proportion of the genome involved in parallel marine–freshwater differentiation that would have led to an overestimation of *λ*, especially in the three-spined stickleback (iterations stopped when no more or no less significant genomic regions were found compared to the previous iteration). Whenever *P* value inflation was present (*λ* > 1) all observed −log_10_(*P*) were divided by *λ* (prior to FDR), thus ensuring that no residual *P* value inflation would exist in the data, also known as GC. For instance, *λ* = 2 (high *P* value inflation) means that a test with −log_10_(*P*) = 10^−2^ after GC correction is no longer significant (10^−2^/*λ* = 0.1)*.* Note that GC was seldom necessary when relatedness was accounted for and GC is not possible when permutation is used to control for multiplicity (Li et al. 2018).

All association analyses were corrected for *P* value inflation and multiplicity using the following four approaches: (1) including relatedness as a random effect and using FDR (“A + FDR”), (2) including relatedness as a random effect and controlling for multiplicity by permutation (“A + perm”), (3) ignoring relatedness and instead using GC to control *P* value inflation, followed by FDR (“GC + FDR”), and (4) not including relatedness as a random effect but controlling for multiplicity using permutation (“perm”; with no possibility for GC).

Due to the two highly divergent lineages of nine-spined sticklebacks in the Atlantic (with some individuals being a result of admixture between them), we included lineage (WL, EL, or ADMIXED) as a cofactor in the analyses for this species. This greatly reduced initial *P* value inflation that otherwise would have caused many false positives or significantly reduced power to detect true significant associations following GC.

### Assessing Sensitivity of Association Analyses to Parameter Settings

With three parameters for defining LD clusters (*|E|*_min_, SNP_min_, and Cor_th_) and four methods to correct for multiplicity and *P* value inflation, the three- and nine-spined stickleback data sets were subjected to a total of 144 tests each. It is important to note, however, that all tests are applied to exactly the same data sets (i.e., tests within species are per definition not independent), such that the cumulative number of significant genomic regions found is quickly expected to reach an asymptote as more parameter combinations are tested, as shown in supplementary figure 3, Supplementary Material online.

A genomic region was considered significantly associated with marine–freshwater differentiation when at least ten unique loci from clusters significant at *a* = 0.05 (after corrections) were also physically clustered in the genome as determined by single-linkage clustering with a distance threshold of 500 kb. All significant clusters from any of the 144 parameter/correction method combinations were included but most loci were found in LD clusters in multiple such combinations. Based on this, we calculated a consistency score *C* for each putative outlier region, denoting the proportion of tests where a given genomic region was found significant, with *C *=* *1 indicating that a given region was significant in all 144 tests. Conversely, low *C*-scores are expected for outlier regions that are only detected in a restricted set of parameter/correction combinations. Note, however, that *C*-scores do not necessarily imply small effect sizes. Our wide range of parameter combinations was necessary to minimize the dependence between parameter values and the number of outlier regions detected in three- and nine-spined sticklebacks, despite vastly different levels of population structuring and potentially fundamentally different mechanisms underlying marine–freshwater parallelism. We deemed outlier regions with *C < *0.05 to be too sensitive to parameter settings to be considered further in downstream analyses (Supplementary fig. 3, Supplementary Material online).

### Regional Parallelism

We also performed EMMAX analyses separately for the geographic regions with large sample sizes of freshwater individuals for both species: Baltic Sea (18, 13), North Sea (20, 23), Norwegian Sea (21, 23), and White and Barents Seas (31, 38), with numbers in brackets indicating sample sizes for three- and nine-spined sticklebacks, respectively. The corresponding marine samples sizes were more variable for both species and were lacking altogether from the Norwegian Sea region for the nine-spined sticklebacks. However, for any genomic region associated with marine–freshwater parallelism, the expectation is that freshwater adapted alleles/haplotypes are found in high frequency in the freshwater populations where they are locally adapted. Conversely, in marine populations, we expect the low frequency of these alleles/haplotypes, regardless of geographic location (in contrast to neutral loci). Thus, to analyze and compare regional parallelism for both species fairly, we pooled all marine samples and contrasted them against freshwater samples from one of the four geographic regions at a time. These analyses were performed on SMLAs based on all unique loci from significant LD clusters that mapped to each significant genomic region identified above. Because the power to detect significant associations depends on the data set, we only compared correlation coefficients as proxies for effect sizes, both when assuming all individuals being unrelated (cor_unrl_) and when including ***A*** as a random effect (cor_A_). This allowed us to assess which geographic regions contributed, and how much, for the overall marine–freshwater differentiation for each significant genomic region.

## Results

### Genetic Variation within Populations

**Fig. 1.**
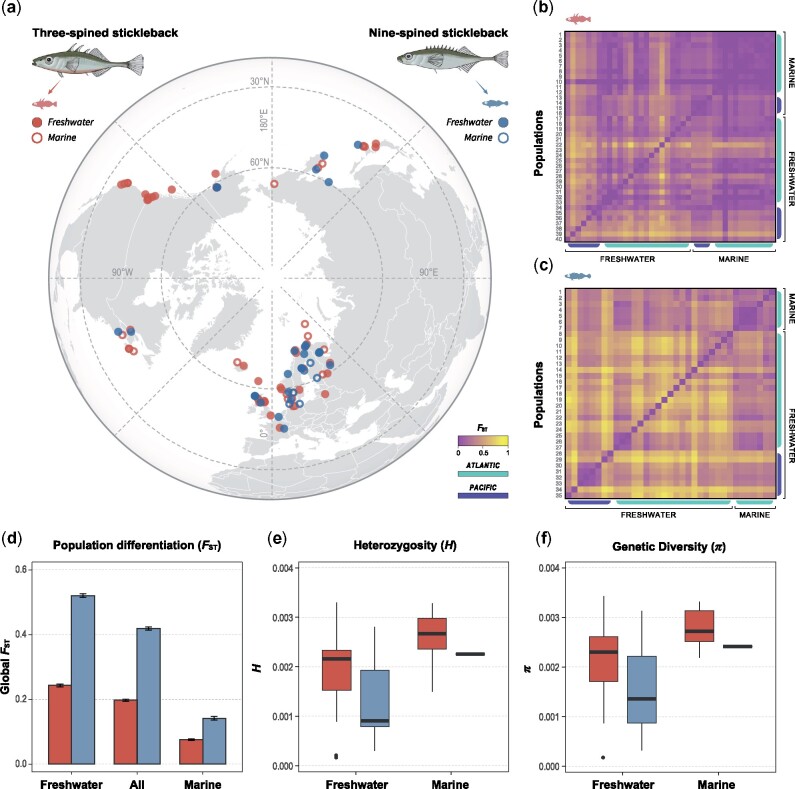
Three-spined sticklebacks exhibit higher levels of genetic variation and lower degree of population differentiation than nine-spined sticklebacks. (*a*) Map showing the species-specific sampling locations. (*b*, *c*) Pairwise population differentiation (*F*_ST_) of three- and nine-spined sticklebacks, respectively. Populations are specified in supplementary table 3, Supplementary Material online. (*d*) Global *F*_ST_ of the two species. Error bars indicate 95% bootstrap CIs. (*e*, *f*) Boxplots of Heterozygosity (*H*) and nucleotide diversity (*π*). Admixed populations in nine-spined stickleback are excluded in (*e*, *f*). See supplementary figure 4, Supplementary Material online, for the estimates of Watterson’s theta (*θ*) and the effect of admixed populations on measures of genetic diversity. GLMM revealed significant differences in genetic diversity (*e*, *f*) between species and ecotypes (see Results).

There were significant differences in levels of genetic diversity between the two species and habitats. Generalized linear mixed model (GLMM) show that average heterozygosity (*H*) was significantly higher in three- than in nine-spined sticklebacks (GLMM: *F*_1,258.85_ = 91.33, *P *<* *0.001; fig. 1*e*). Marine populations harbored higher heterozygosity than freshwater populations in both species (GLMM: *F*_1,257.14_ = 25.70, *P *<* *0.001; fig. 1*e*). Both nucleotide diversity (*π*, Nei and Li 1979) and Watterson’s *θ* (Watterson 1975) were higher in the three- than in the nine-spined stickleback populations (*π*: GLMM, *F*_1,58.91_ = 10.34, *P *=* *0.002, fig. 1*f*;*θ*: GLMM, *F*_1,58.98_ = 12.48, *P *<* *0.001, supplementary fig. 4*a*, Supplementary Material online), and higher in marine populations than in freshwater populations (*π*: GLMM, *F*_1,58.98_ = 12.49, *P *<* *0.001, fig. 1*f* and *g*; *θ*: GLMM, *F*_1,58.61_=7.25, *P *<* *0.01, supplementary fig. 4*a*, Supplementary Material online). Species × habitat interactions were not significant in any of the analyses.

When incorporating nine-spined stickleback populations showing strong signatures of admixture (see Materials and Methods) in the analyses, the differences in genetic diversity (*H*, *π*, and *θ*) between habitats were still significant (*H*: GLMM: *F*_1,320.45_ = 54.59, *P *<* *0.001; *π*: GLMM, *F*_1,70.09_ = 9.24, *P *=* *0.003; *θ*: GLMM, *F*_1,69.93_ = 8.21, *P *=* *0.005; supplementary fig. 4*b*–*d*, Supplementary Material online), but those between species were no longer significant (supplementary fig. 4*b*–*d*, Supplementary Material online). This indicates that admixture has had a significant positive effect on genetic diversity in the admixed nine-spined stickleback populations. Indeed, admixed populations have significantly higher heterozygosity than nonadmixed populations (GLMM, *F*_1,320.02_ = 75.82, *P *<* *0.001; supplementary fig. 4*e*, Supplementary Material online).

### Genetic Differentiation among Populations

**Fig. 2.**
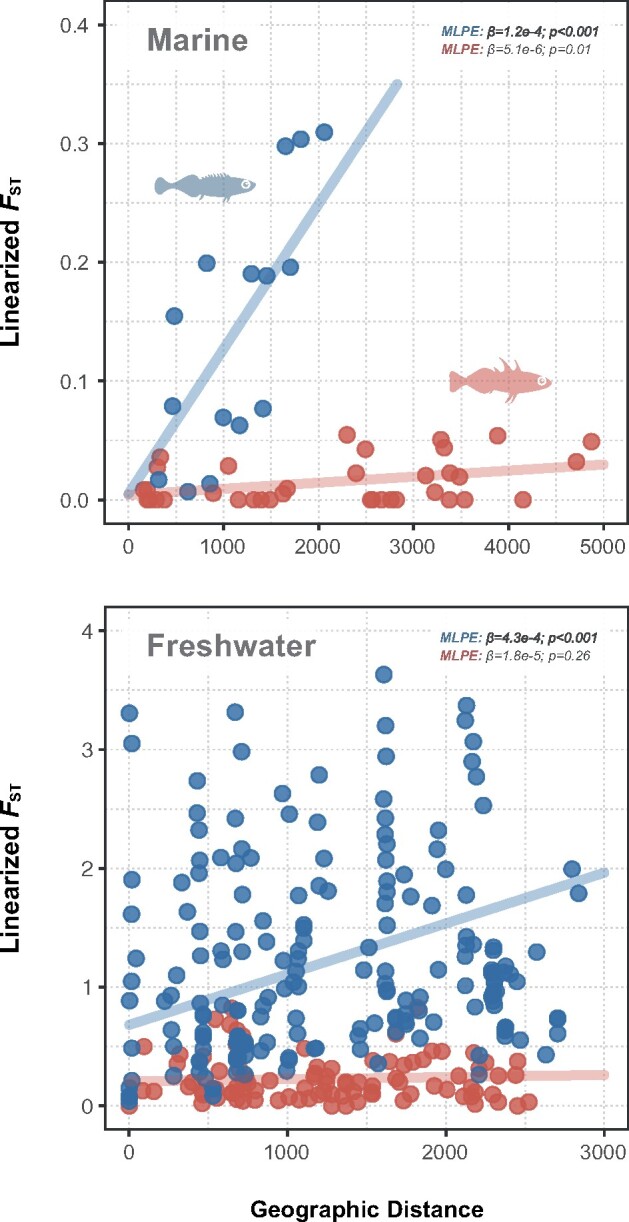
Isolation-by-distance (IBD) was tested across (*a*) marine and (*b*) freshwater three- (red) and nine-spined stickleback (blue) populations using MLPE model. The slope of the regression coefficients (*β*) suggests stronger IBD in nine- than in three-spined sticklebacks. IBD comparisons were restricted to European populations (see Materials and Methods).

The degree of genetic differentiation, measured by Weir and Cockerham *F*_ST_ (Weir and Cockerham 1984) was significantly higher among nine-spined stickleback populations (global *F*_ST_ = 0.419, 95% CI: 0.414–0.424; fig. 1*d*) than three-spined stickleback populations (global *F*_ST_ = 0.198; 95% CI: 0.194–0.201; fig. 1*d*). This was true also when only considering populations from the same genetic clades in nine-spined sticklebacks (supplementary fig. 5, Supplementary Material online). In both species, there was less differentiation among marine than freshwater populations (fig. 1*d*). Furthermore, maximum‐likelihood population effects (MLPE) mixed models show significant Isolation by Distance (IBD) for the nine-spined stickleback in both marine and freshwater environments (MLPE, *P *≤* *0.01; fig. 2). In the three-spined stickleback, IBD was significant in marine (MLPE, *P *<* *0.001; fig. 2) but not in freshwater habitats (MLPE, *P *<* *0.26; fig. 2). A comparison of the IBD slopes (MLPE regression coefficient *β*) revealed that the IBD in the marine habitat was 23.9 times stronger in the nine-spined than in the three-spined sticklebacks (MLPE, *β*  =  1.2e−4 vs. 5.1e−6; fig. 2).

### Phylogenetic Histories

**Fig. 3.**
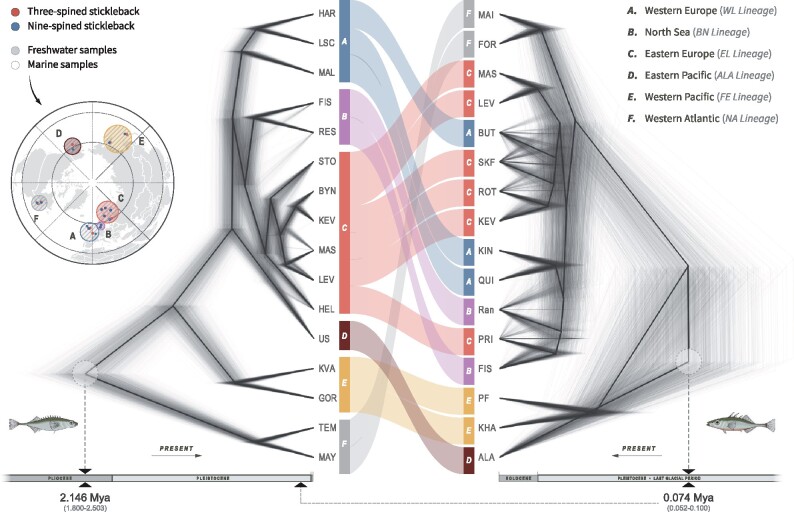
Time-calibrated phylogenies of three- and nine-spined sticklebacks inferred with SNAPP. The topology and divergence times of phylogenetic trees of nine- (left) and three-spined sticklebacks (right) are presented and compared using populations across the same, or geographically close, sampling sites. Colors correspond to the different lineages (A–F) of nine-spined sticklebacks. Arrows in the bottom indicate directions of timeline from past to present. The TMRCA of three-spined sticklebacks is marked in the timeline of the nine-spined sticklebacks for comparison. Population identifiers were simplified for clarity; see supplementary figure 9*e*, Supplementary Material online, for a map of sampled populations. The maximum-clade-credibility summary trees of the SNAPP phylogenies indicating divergence times and calibration points are given in the supplementary figure 6, Supplementary Material online.

The comparison of the time-calibrated phylogenies between three- and nine-spined sticklebacks (fig. 3) revealed contrasting phylogenetic relationships and colonization histories across their global distribution. The time to most recent common ancestor (TMRCA) of all lineages of nine-spined sticklebacks was 2.146 million years ago (Ma) in late Pliocene (95% HPD interval [hereafter in parenthesis]: 1.800–2.503 Ma), which is much older than the TMRCA of the three-spined stickleback 0.074 Ma in late Pleistocene (0.052–0.100 Ma).

The most basal lineage of nine-spined sticklebacks was from the Western Atlantic (F in fig. 3). In contrast, the Western Atlantic clade of three-spined sticklebacks was among the most recently diverged lineages in line with earlier findings (Fang et al. 2018, 2020b). The most basal lineage in three-spined sticklebacks was the Eastern Pacific clade (D in fig. 3), whereas the nine-spined sticklebacks from this area were more recently diverged (ALA lineage) with a divergence time close to the TMRCA of its European lineages (0.766 Ma [0.644–0.887 Ma]; fig. 3 and supplementary fig. 6, Supplementary Material online).

The European three-spined stickleback populations have diverged recently (A, B, and C in fig. 3; 0.026 Ma [0.018–0.035 Ma]), and show clear signs of incomplete lineage sorting. In contrast, the European nine-spined stickleback populations had deep and clear lineage separation (three lineages [A, B, and C] diverged 0.762 Ma [0.638–0.882 Ma]), with evidence for introgression between the Eastern European (B) and the North Sea (C) lineages (fig. 3; Feng et al. 2020).

### Patterns of Genetic Parallelism

**Fig. 4.**
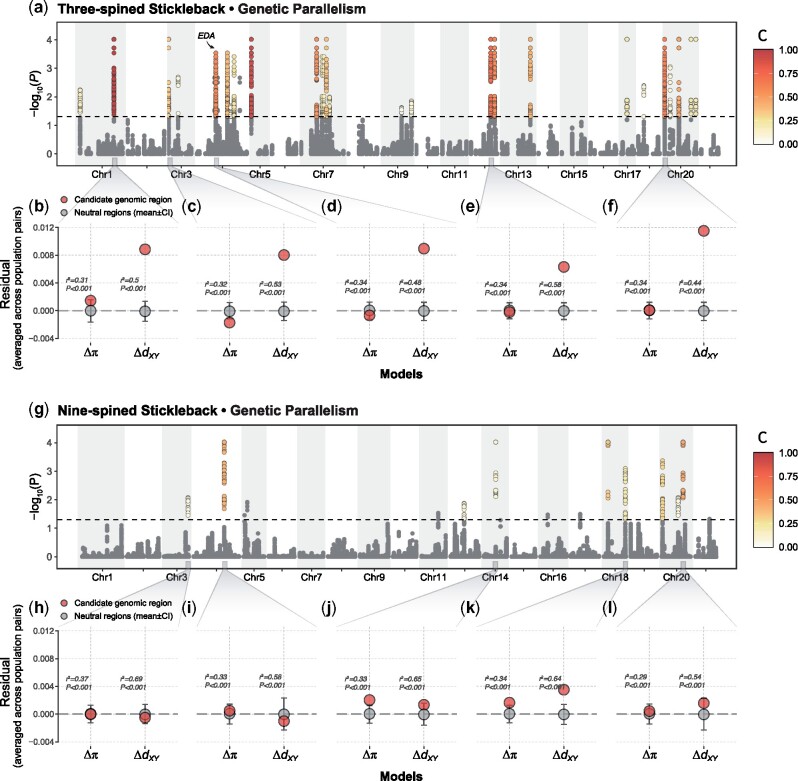
Three-spined sticklebacks exhibit stronger levels of parallel genetic evolution than nine-spined sticklebacks. (*a*, *g*) Manhattan plots of −log_10_(*P*) value testing for associations between LD clusters and ecotype (EMMAX). Color for each unique genomic region indicates the proportion of all combinations of parameter/correction method settings a given region was found significant in (*C*-score) after corrections for multiple testing and *P* value inflation (gray indicates *C*-score < 0.05). (*b*–*f*, *h*–*l*) Summaries of residuals of linear regression models based on the genetic diversity (*π*) and genetic divergence (*d_XY_*) derived from marine–freshwater population pairs for selected outlier regions (see Materials and Methods). The squared correlation coefficient (*r*^2^) is shown as an averaged value across all models from different population pairs. Summaries for all regions are given in supplementary figure 4, Supplementary Material online. All models were statistically significant (*P *<* *0.001).

**Fig. 5.**
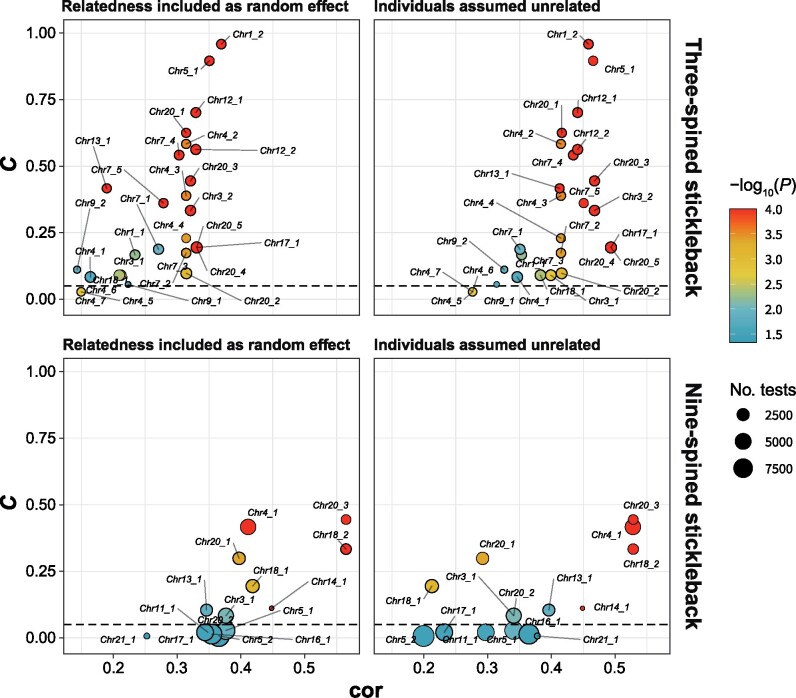
Relationship between *C*-score and effect size (cor). Figure depicts *C* as a function of effect size (cor; 95% quantile across all significant LD clusters mapping to a given genomic region) for outlier genomic regions, when (left panel) relatedness is accounted for and (right panel) when individuals are assumed to be unrelated for three- and nine-spined sticklebacks (upper and lower panels, respectively). Size indicates the mean number of tests for significant LD clusters for a given region (a function of *|E|*_min_, SNP_min_, and Cor_th_, see main text for details), with smaller numbers indicating that a region is only significant when fewer and larger LD clusters are tested and color indicates the most significant *P* value across any correction method (“A + FDR,” “A + perm,” “GC + FDR,” and “perm”). Only outlier regions above the horizontal dashed line (*C *>* *0.05) are considered in our analyses.

LDna and LMMs were used to detect genomic regions with high marine–freshwater differentiation in three- and nine-spined sticklebacks. When including relatedness as a random effect in association tests between SMLAs and ecotype, *P* value inflation, *λ*, was reduced from *λ* = 1.95 to *λ* =1.01 and from *λ* = 1.73 to *λ* = 1.32 for three- and nine-spined sticklebacks, respectively. Thus, accounting for relatedness reduced *P* value inflation completely in three-spined sticklebacks, but not in nine-spined sticklebacks. Nevertheless, GC ensured that any residual *P* value inflation was accounted for except when using permutation for which GC was not possible. After corrections for *P* value inflation and multiplicity, the number of outlier regions that were significant in at least 5% of all parameter combinations/correction methods (*C *>* *0.05) was 26 for three-spined sticklebacks and nine for nine-spined sticklebacks (figs. 4 and 5). Although no single parameter combination detected all these outlier regions, for three-spined sticklebacks the parameter settings that detected the most significant regions were: *|E|*_min_*=* 10, Cor_th_*=* 0.5, and any combination of SNP_min_*=* [10, 20] (detecting all but the two ChrIX outlier regions), with the corresponding parameter settings for nine-spined sticklebacks being: *|E|*_min_ = 20, SNP_min_ = 10, and any combination of Cor_th_ = [0.6, 0.7, 0.8] (detecting all but the ChrXIV outlier region). However, no outlier regions in nine-spined sticklebacks were found in >50% (*C *>* *0.5) of the parameter combinations, while this was the case for seven outlier regions in three-spined sticklebacks (fig. 5 and supplementary table 1, Supplementary Material online), with the corresponding numbers for *C *>* *0.25 being 13 and 4, respectively. Thus, regardless of *C*-score, the number of outlier regions detected was always larger in three- than in nine-spined sticklebacks, showing that outlier detection in nine-spined sticklebacks was more dependent on parameter settings than that in three-spined sticklebacks. As a consequence, widely different results could have been obtained, particularly for nine-spined sticklebacks, if only a single (arbitrary) parameter setting had been chosen for LDna.

Although all outlier regions with high *C*-scores tended to also have large effect sizes (cor; estimated from SMLAs based on all significant loci from a given outlier region, see Materials and Methods; fig. 5), some outlier regions with large effect sizes (e.g., Chr17_1, Chr20_4, and Chr20_5 for three-spined sticklebacks and Chr14_1 for nine-spined sticklebacks) did not have high *C*-scores (fig. 5). These regions were more sensitive to parameter settings, but when they were detected, they tended nevertheless to have large effect sizes (and be highly significant). The Chr14_1 (*C *=* *0.11) outlier region in nine-spined sticklebacks, for instance (cor = 0.45, both when correcting and not correcting for relatedness), was only detected when *|E|*_min_ = 40 and SNP_min_ =40.

The mean size for outlier regions was 3.0 times larger for three-spined sticklebacks compared with nine-spined sticklebacks (154 kbps vs. 52 kbps; supplementary table 1, Supplementary Material online), with the mean number of unique significant loci mapping to each region being 2.7 times larger in three- compared with nine-spined sticklebacks (115 SNPs with a range of 10–1,341 vs. 42 SNPs with a range of 10–109). The total number of loci from significant LD clusters mapping to outlier regions was 379 and 2,996, for three-spined and nine-spined sticklebacks, respectively. However, a large proportion of the loci (43%) in three-spined sticklebacks mapped to the Chr1 inversion.

**Fig. 6.**
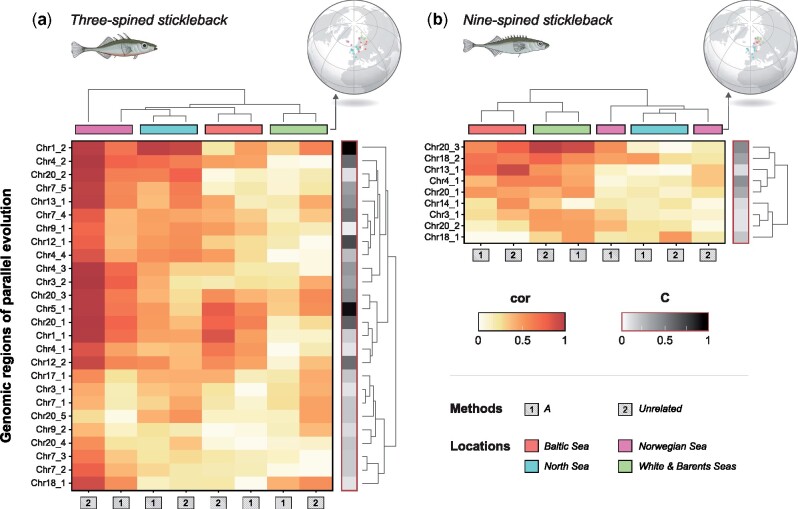
Regional parallelism. Shown are heatmaps of effect sizes (cor) from linear regressions between SMLAs and ecotype (EMMAX) for outlier regions (*C*-score ≥ 0.05) separately for four geographic regions. Results are shown for (*a*) three- and (*b*) nine-spined sticklebacks, when assuming individuals are unrelated (unrl) and when a relatedness matrix (***A***) was included as a random effect. Gray side bars indicate the *C*-score.

Focusing on four different geographic regions within the Atlantic Ocean, the mean effect size across all genomic regions was cor_unrl_ = 0.38 (SD = 0.24) and cor_A_ = 0.28 (SD = 0.17) for three-spined sticklebacks and cor_unrl_ = 0.29 (SD = 0.20) and cor_A_ = 0.27 (SD = 0.17) for nine-spined stickleback. No outlier region associated with marine–freshwater parallelism displayed universally high effect sizes across all analyzed geographic regions in neither of the species (fig. 6). The geographic region with the highest mean effect size across the outlier regions for three-spined sticklebacks was the Norwegian Sea (cor_unrl_ = 0.38, SD = 0.17; cor_A_ = 0.66, SD = 0.2), with least evidence for parallelism being found in White and Barents Sea (cor_unrl_ = 0.24, SD = 0.15; cor_A_ = 0.14, SD = 0.078) and in the Baltic Sea regions (cor_unrl_ = 0.294, SD = 0.19; cor_A_ = 0.22, SD = 0.14). In contrast, the two geographic regions with highest effect sizes across all outlier genomic regions in nine-spined sticklebacks were the opposite of three-spined sticklebacks, namely White and Barents Sea (cor_unrl_ = 0.43, SD = 0.17; cor_A_ = 0.39, SD = 0.17) and the Baltic Sea regions (cor_unrl_ = 0.41, SD = 0.22; cor_A_ = 0.42, SD = 0.19).

The genomic regions putatively involved in parallel evolution in three-spined sticklebacks show an excess in absolute divergence (Δ*d_XY_*) compared with neutral genomic regions, suggesting the ancient origin of the selected regions (as per Nelson and Cresko 2018; supplementary method 8, Supplementary Material online). On the contrary, the genomic regions putatively involved in parallel evolution in nine-spined sticklebacks do not show significant Δ*d_XY_*. Specifically, in three-spined sticklebacks, 16 out of 26 genomic regions under selection had higher Δ*d_XY_* than the 95% CI of Δ*d_XY_* estimated from 100 random “neutral” regions across the genome l (fig. 4*a*–*d* and supplementary fig. 7, Supplementary Material online). Those ancient regions included EDA gene and the inversion in Chr1 (Jones et al. 2012; Fang et al. 2020; fig. 4*b* and *d*). In contrast, only one out of nine regions under selection in nine-spined sticklebacks showed (slightly) higher Δ*d_XY_*, exceeding the 95% CI of Δ*d_XY_* for neutral regions (fig. 4*k*). Full results of genetic diversity and divergence in the candidate genomic regions were given in supplementary figure 7, Supplementary Material online.

There was a significant negative correlation between divergence times and the level genetic parallelism in nine-spined sticklebacks (MLPE: *β* = −2e−4, *P *<* *0.001; supplementary fig. 8, Supplementary Material online). However, there was no significant correlation in three-spined sticklebacks (MLPE: *β* = −0.014, *P *=* *0.76; supplementary fig. 8, Supplementary Material online).

## Discussion

Theory predicts the probability of parallel evolution to be negatively correlated with divergence time and positively correlated with effective population size and population connectivity (MacPherson and Nuismer 2017). Despite their similar life histories, ecologies, and distribution ranges, three- and nine-spined sticklebacks show dramatically different phylogenetic histories, within population genetic diversities and population structuring across comparable geographic distances and as predicted, very different levels of genetic parallelism in response to colonization of freshwater environments. As the results show, gene flow between nine-spined stickleback populations is more restricted than between three-spined stickleback populations, resulting in a more heterogeneous pool of SGV available for freshwater adaptation, thereby reducing the probability of parallel evolution in the former. These findings indicate that the two species differ markedly in the fundamental processes affecting the distribution of adaptive genetic variation among their demes.

Three-spined sticklebacks displayed a larger number (2.9 times) of genomic regions involved in marine–freshwater parallelism than nine-spined sticklebacks, and these regions were on average three times larger and harbored eight times more loci. Most outlier regions in three-spined sticklebacks show excess divergence (Δ*d_XY_*), a result in line with evidence from other studies (Nelson and Cresko 2018) suggesting ancient origins of haplotypes involved in parallel evolution in this species. In contrast, the outlier regions in nine-spined sticklebacks do not follow the same pattern and the mostly nonsignificant Δ*d_XY_* values suggests young age of their haplotypes. Furthermore, for both species, no marine–freshwater divergence associated region showed high effect sizes across all studied geographic regions, suggesting that parallelism is often geographically limited. Below we discuss how these findings shed new light on our understanding of how geographic patterns of parallel local adaptation is shaped by demographic and phylogenetic history.

### Genetic Diversity, Population Connectivity, and Phylogenetic History

Genetic diversity was generally lower for nine- than three-spined sticklebacks and in freshwater compared with marine populations. These findings align with earlier studies showing lower genetic diversity in nine- than in three-spined stickleback populations (DeFaveri et al. 2012; Merilä 2013a,b), and in freshwater fish populations than marine fish populations in general (Avise et al. 1987; Ward et al. 1992, 1994; DeWoody and Avise 2000; McCusker and Bentzen 2010). Assuming that three-and nine-spined sticklebacks exhibit similar mutation rates, the observed differences in *θ* (three-spined > nine-spined stickleback) should reflect differences in coalescent *N*_e_ (as *θ *= 4 *N*_e_*μ*). Hence, lower *N_e_*, stronger population structure and stronger IBD all contribute to more heterogeneous pools of SGV in nine-spined than in three-spined sticklebacks. Assuming that adaptive genetic variation follows the same general pattern, SGV for freshwater adaptation is expected to be considerably reduced in nine- compared with three-spined sticklebacks.

Our analyses revealed that the nine-spined stickleback lineages were far older than three-spined stickleback lineages, and that the degree of genetic parallelism decreased as a function of divergence time in the nine-spined stickleback. All this supports the notion that differences in divergence time influence the probability of parallel evolution, as pools of SVG get increasingly differentiated with time (MacPherson and Nuismer 2017). However, the question of which is a more important determinant of the probability of parallel evolution—divergence time or gene flow—is not easily answered because the two are not independent: restricted gene flow is a prerequisite for the formation and maintenance of distinct lineages.

### Geographic Heterogeneity in Selection Optima?

Parallel evolution not only requires access to the same pool of SGV (Barrett and Schluter 2008; Schluter and Conte 2009) but also parallelism of selection optima across the distribution range, which cannot be taken for granted (Stuart et al. 2017; Bolnick et al. 2018; Magalhaes et al. 2021). Thus, lower parallelism in selection optima across freshwater habitats could also explain the lower degree of parallelism in nine- than three-spined sticklebacks. However, geographic differences in SGV can result in contrasting patterns of genetic parallelism, both globally (Fang et al. 2020) and locally (Leinonen et al. 2012). Based on a small subset of the data used here, simulations in Kemppainen et al. (2021) show that the level of IBD characteristic of nine-spined stickleback populations (as opposed to three-spined stickleback-like scenarios) is sufficient to severely restrict SGV for local adaptation. Thus, although parallel angles of selection are a prerequisite, parallel evolution also relies on access to the necessary ancestral SGV for local adaptation. That ancestral SGV in turn is determined by population demographic parameters such as *N*_e_ and population connectivity.

The reported differences in the degree of parallel evolution between two stickleback species could be argued to be an artifact of the inherent difficulty of detecting outlier loci among highly differentiated populations (Hoban et al. 2016; Matthey‐Doret and Whitlock 2019; Galloway et al. 2020). However, whenever background differentiation is high (due to population structuring), we can also expect *P* value inflation due to relatedness. Accounting for relatedness can in some circumstances be expected to increase statistical power to detect outliers (Kang et al. 2008, 2010). For instance, when multiple populations in similar habitats display high frequencies of the same genetic variants (i.e., parallel evolution), the more divergent the populations are, the stronger the contrast between neutral genetic background (reflecting relatedness) and genomic regions under selection will be. Because neither effect sizes nor *P* value inflation (both of which are important determinants of statistical power) differed much between nine-and three-spined sticklebacks for the outlier regions, there is no reason to doubt the conclusion that the marine–freshwater differentiated regions in three-spined sticklebacks outnumber such regions in nine-spined sticklebacks across a wide range of parameter/threshold values and correction methods. This and other methodological considerations are discussed further in supplementary information 1, Supplementary Material online.

### Geographic Heterogeneity in Marine–Freshwater Parallelism

Historically, much of the parallel evolution research in three-spined sticklebacks has focused on Eastern Pacific populations (Nelson and Cresko 2018; Hohenlohe and Magalhaes 2019; Fang et al. 2020). However, it is becoming increasingly clear that parallelism in three-spined sticklebacks is not as universal and global as previously thought (Terekhanova et al. 2019; Fang et al. 2020). This is also clear from our analyses (with more extensive geographic coverage than in previous studies), where heterogeneity in effect sizes across the different geographic region was found not only in nine- (where this was expected) but also in three-spined sticklebacks. In our analyses of genetic parallelism, we limited our comparisons to the Atlantic region as we lacked samples of marine nine-spined sticklebacks from the Eastern and Western Pacific regions. Therefore, we do not know if the patterns seen in the Atlantic region can be generalized to the rest of the species distribution range. However, there is a good reason to believe that inclusion of populations from the Eastern Pacific would have revealed stronger, not weaker, differences in levels of parallel evolution between the two taxa. Namely, the extent of genetic parallelism in the three-spined sticklebacks from the Pacific region is far stronger than that in the Atlantic (Fang et al. 2020). Although this might at first suggest that inclusion of Eastern Pacific samples to the comparison might recover more genetic parallelism also in the nine-spined sticklebacks, we believe that the opposite is more likely to be true. The Eastern Pacific is the ancestral range of the three-spined stickleback, which harbors most of the SGV involved in marine–freshwater adaptation (Fang et al. 2018; Liu et al. 2018; Terekhanova et al. 2019). In contrast to three-spined sticklebacks, the oldest populations of nine-spined sticklebacks are located in the Western Atlantic region (fig. 3), and therefore, it is more parsimonious to assume that at least a part of the SGV involved in parallel evolution might have been lost during the invasion of the Pacific region. Hence, the inclusion of Pacific populations of both species into the analyses would likely reveal even stronger differences than observed now.

### Age of Freshwater Adapted Alleles

Although nine-spined stickleback populations have a longer evolutionary history than the three-spined stickleback populations, the genomic haplotypes under parallel selection in three-spined sticklebacks appear to be of more ancient origin than the populations in which they are segregating. A possible explanation for this counterintuitive finding may lie in the effect of gene flow and *N*_e_ on the ability of species to maintain ancestral haplotypes in the pool of SGV; the larger *N*_e_ and the weaker population subdivision make this scenario more likely in three- compared with nine-spined sticklebacks. Although the Atlantic three-spined stickleback populations have a relative young evolutionary history (colonization occurred 29.5–226.6 Ka; Fang et al. 2020), the freshwater-adapted alleles in the SGV pool originate from the Eastern Pacific region (Fang et al. 2020), which harbors these ancient haplotypes (∼6 million years old; Nelson and Cresko 2018). In contrast, lower *N_e_* and gene flow in the nine-spined stickleback marine populations are expected to limit the maintenance and geographic spread of SGV. This could lead to higher turnover rates of freshwater adapted alleles, higher dependence on novel mutations and consequently higher geographic heterogeneity in parallel evolution in nine-spined sticklebacks.

### Implications for Local Adaptation

The implications of the absence of a homogenous pool of SGV in the nine-spined stickleback are relevant to local adaptation in general. Populations colonizing new environments may lose potentially beneficial variation via bottlenecks and founder events. With limited or absent gene flow, the lost variability cannot easily be regained. Consistent with this, Kemppainen et al. (2021) demonstrated that the genetic architecture underlying pelvic reduction (a common freshwater adaptation in sticklebacks) is highly heterogeneous in nine-spined sticklebacks even across short (<10 km) geographic distances. In addition, many freshwater populations lacked pelvic reduction altogether, probably because they lacked the SGV underlying this trait, thus restricting the populations to suboptimal phenotypes. A possible example where restricted access to SGV has led to potentially less optimal freshwater adaptation can also be found in three-spined sticklebacks. In a small and isolated region of northern Finland, fully plated three-spined sticklebacks displayed reduced lateral plate height possibly as a compensatory adaptation to the genetic constraint imposed by the lack of the low-plate EDA allele in these populations (Leinonen et al. 2012). Thus, it is important to note that lack of a large pool of SGV not only limits parallel evolution but also local adaptation more generally.

The lack of SGV to fuel local adaptation can be mitigated by introgression between divergent clades as this can substantially increase the genetic variation in the affected populations (Anderson 1949; Arnold 1997; Marques et al. 2019). This appears to be the case for the Baltic Sea nine-spined stickleback populations which have experienced introgression from divergent western European populations (Shikano et al. 2010; Teacher et al. 2011; Feng et al. 2020); a comparison of admixed and nonadmixed populations revealed the former to have significantly higher heterozygosity than the latter. As admixed populations were excluded from the analyses of within population genetic diversity, these did not have any effect on our inference beyond reducing sample sizes available for analyses. Nevertheless, the results demonstrate that introgression is an important determinant of genetic diversity and thus potentially also of the adaptive potential of populations. This is consistent with Baltic Sea populations having large effect sizes for a subset of the regions that have large effect sizes also in the White and Barents Sea possibly compensating for the lack of freshwater-adapted alleles in the Western lineage (which have much lower effect sizes for all outlier regions).

### Genetic Differentiation and Speciation

It is intriguing that the two species of sticklebacks studied here do not display only highly contrasting population structures but come from different genera containing an equally contrasting number of recognized species. Although it is tempting to think that there could be a causal connection between among population connectivity and propensity to speciate, one needs to notice that there is also evidence to suggest that there may be yet undescribed species in both genera (Taylor et al. 2006; Guo et al. 2019). Hence, counting taxonomically valid species as proxies of speciosity can be misleading.

What may be more interesting to consider is the process of speciation. In the case of the three-spined stickleback “species pairs,” speciation seems to progress rapidly toward completion, but full reproductive isolation is not usually reached (McKinnon and Rundle 2002). In fact, whenever the ecological conditions that drove the evolution of reproductive isolation in the first place cease to exist, hybridization and reverse speciation is known to occur (Rudman and Schluter 2016; Marques et al. 2019). Hence, this suggests that strong genetic incompatibilities have not had time to evolve. The marked between species difference in divergence times of local three- and nine-spined populations shown in this study suggest that one should expect speciation through accumulation of genetic incompatibilities to be much more likely in the *Pungitius* than in the *Gasterosteus* genus. There is indeed some evidence for evolution of incompatibilities in *Pungitius* (Natri et al. 2019), and although hybridization has occurred fairly frequently (Guo et al. 2019), the species have not collapsed into hybrid swarms. It is tempting to speculate that the same features that can drive local adaptation and initial progress along the speciation continuum in the short term are actually limiting factors in driving speciation to completion. Hence, the two genera should provide an interesting model system for future studies focused on the roles of adaptive divergence versus incompatibilities in generating new species.

In conclusion, the results establish that the two codistributed stickleback species possess strikingly different population genetic structures suggesting far more limited gene flow and, hence, a more heterogeneous pool of SGV among the nine-spined, than among the three-spined stickleback populations. This greater heterogeneity likely underlies the observed lower degree of genetic parallelism among the nine-spined stickleback populations. Furthermore, there appears to be generally less SGV in the nine- than in the three-spined stickleback possibly because of lower long-term effective population sizes of the latter. However, high levels of SGV were detected in those few nine-spined stickleback populations where introgression from other related lineages or species has been documented. All in all, the results suggest that because of the contrasting levels of heterogeneity in SVG, the two stickleback species are differently disposed to adapt to similar selection pressures via parallel and nonparallel genetic mechanisms.

## Materials and Methods

### The Study Species

The two study species are ecologically very similar and are frequently syntopic in both marine and freshwater habitats (e.g., Copp and Kovac 2003; Ojaveer et al. 2003; DeFaveri et al. 2012; Raeymaekers et al. 2017). However, there is a tendency for the three-spined stickleback to be more common in marine habitats, and for the nine-spined stickleback to be more common in freshwater habitats (Wootton 1976, 1984). Both species are small (typically < 100 mm), with similar lifespans (Baker 1994; DeFaveri and Merilä 2013; DeFaveri et al. 2014) and breeding habits (Wootton 1976, 1984), and exhibit male parental care; males build and attend nests in which multiple females can lay their eggs (Wootton 1976, 1984). Females of both species can lay single or multiple clutches of ca. 100–500 eggs per breeding season (Wootton 1976, 1984; Baker 1994; Heins and Baker 2003; Herczeg et al. 2010). Hence, we do not expect a large variation in levels of SGV between the two species due to differences in life-history traits (cf. Romiguier et al. 2014; Ellegren and Galtier 2016).

### Sample Collection and Sequencing

The data set is composed of 166 (47 marine and 119 freshwater) three-spined stickleback and 181 (48 marine and 133 freshwater) nine-spined stickleback individuals. The 166 three-spined stickleback samples were the same as used in Fang et al. (2020). Data for 75 nine-spined stickleback samples were retrieved from an earlier Restriction Site Associated DNA (RAD) sequencing study (Guo et al. 2019) and a whole-genome resequencing (WGRS) study (Feng et al. 2020). New samples of nine-spined sticklebacks were sequenced specifically for this study, including 23 samples using RAD sequencing (protocol following Guo et al. 2019, using the PstI enzyme) and 83 samples using WGRS at 10× coverage (protocol following Feng et al. 2020). In total, 63 populations (26 marine and 37 freshwater; sample sizes: 1–10) of three-spined stickleback and 36 populations (7 marine and 29 freshwater populations; sample sizes: 1–20) of nine-spined stickleback were included in this study. The sampling sites are shown in fig. 1*a* and detailed sample information (population acronyms, sample sizes, lineages, sampling site coordinates, sequencing information, etc.) are given in supplementary table 2, Supplementary Material online.

According to the known phylogenetic relationships within each species, the three-spined stickleback samples were assigned to seven lineages: Eastern Pacific (EP), Western Pacific (WP), Western Atlantic (WA), White and Barents Seas (WB), North Sea and British Isles (NS), Baltic Sea (BS), and Norwegian Sea (NOR) (Fang et al. 2018); and the nine-spined stickleback samples were assigned to six lineages; Western Europe (WL), Eastern Europe (EL), Baltic and North Seas (BN; this lineage has been formed through admixture between the Western European [WL] and Eastern European [EL] lineages; Teacher et al. 2011; Guo et al. 2019; Feng et al. 2020), Far East (FE), North America (NA), and Alaska (ALA).

The new stickleback samples were collected during the local breeding seasons with seine nets and minnow traps. After euthanizing the fish with an overdose of MS-222, whole fish or fin clips were preserved in ethanol for DNA-extractions using the salting-out method (Sunnucks and Hales 1996). The sample collection in Finland was conducted with personal fishing licenses and permissions from the landowners according to the Finnish Fishing Law (5§ 27.5.2011/600). In other countries, the sampling was performed under respective national licenses granted to the sample providers. The study does not involve animal experiments according to the Finnish National Animal Experiment Board (#STH379A and #STH223A). For the newly sequenced nine-spined stickleback samples, the RAD-sequencing data and the WGRS data were obtained with the protocols given in Guo et al. (2019) and Feng et al. (2020), respectively.

### Genotype Likelihood Estimation

The same bioinformatics pipelines were applied to both species. For each species, all RAD and WGRS sequences were mapped to their respective reference genomes with BWA mem v.0.7.17 (Li and Durbin 2010). The reference genome of the three-spined stickleback was retrieved from the Ensembl database (release-92; Yates et al. 2020) and that of the nine-spined stickleback from (Varadharajan et al. (2019). Genotype likelihoods were estimated from the mapped reads using ANGSD v.0.93 (Korneliussen et al. 2014) with the same parameter settings for both species. Quality filtering parameters are explained in detail in supplementary method 5, Supplementary Material online. The raw output of genotype likelihoods from the 166 three-spined sticklebacks comprised 2,511,922 SNPs and those of the 181 nine-spined sticklebacks 7,938,802 SNPs. The difference in SNP numbers between species partly reflects the larger proportion of WGS samples in the latter species (80.1%) than in the former species (22.9%).

### Genetic Diversity and Differentiation

Genetic diversity within populations was estimated with ANGSD and custom R-scripts (supplementary method 6, Supplementary Material online; Momigliano et al. 2021). We computed population nucleotide diversity (*π*), Watterson’s *θ* (*θ *= 4 *N*_e_*μ*, where *N*_e_ is effective population size and *μ* the mutation rate) and individual heterozygosity (*H*, the proportion of heterozygous sites within an individual genome). As some of the sampled populations are known to be admixed (Guo et al. 2019; Feng et al. 2020; supplementary method 6, Supplementary Material online), their genetic diversity was expected to be elevated. We report the results both when excluding and including the admixed nine-spined stickleback populations. In each species, allelic differentiation *F*_ST_ (Weir and Cockerham 1984) was calculated over all samples, within marine and within freshwater ecotypes (global *F*_ST_ over all loci), and between populations (pairwise *F*_ST_). To do so, we used a subset of high-quality genotypes to estimate global *F*_ST_ for each ecotype and the pairwise *F*_ST_ between all populations with the R packages *hierfstat* (Goudet 2005) and *StAMPP* (Pembleton et al. 2013), respectively. Details of the methods used to estimate genetic diversity and differentiation are specified in supplementary method 6, Supplementary Material online.

The levels of genetic diversity (*H*, *π*, and *θ)* between the two species were compared by fitting generalized linear mixed-effects models (GLMMs) in R using the packages *lme4* (Bates et al. 2014) and *Car* (Fox et al. 2012). The models treat species, habitat (freshwater and marine) and their interaction as fixed effects. The geographic region was set as a random effect to account for nonindependence between populations across regions. Nonsignificant interactions were deleted from the final models. To test differences in global *F*_ST_ between species and habitats, we performed bootstrapping based on 10,000 resampled data sets in which each resample consisted of one-third of the markers and one-third of the samples to obtain the 95% CIs.

### Isolation-by-Distance

Tests for IBD were performed by regressing pairwise genetic distances (linearized *F*_ST_ = *F*_ST_/[1 − *F*_ST_]; Slatkin 1995) against pairwise geographic distances between populations. Our sampling of the nine-spined sticklebacks from the Eastern Pacific region was very thin: only two freshwater and no marine populations were sampled. Therefore, to characterize IBD for different ecotypes (marine and freshwater populations), we performed the IBD tests on the European populations (see supplementary fig. 9*d*, Supplementary Material online, for sampling map), where both ecotypes for both species were available. Geographic distances were measured between marine populations based on the pairwise least-cost geographic distances across marine environments using the R Package *Marmap* (Pante and Simon-Bouhet 2013), and between freshwater populations based on world geodetic system with the R package *raster* (Hijmans and van Etten 2014). To test if the IBD relationships differed between the two stickleback species and habitats, the IBD regressions were fitted with MLPE models to account for the nonindependence of pairwise distances (Clarke et al. 2002) and slopes of the IBD regressions for both species were compared. The MLPE analyses were performed using the R packages *corMLPE* (Clarke et al. 2002) and *nlme* (Pinheiro et al. 2017).

The White Sea marine population of the nine-spined stickleback (RUS-LEV) has a close phylogenetic relationship with the marine populations of the Baltic Sea, because the latter originated from a postglacial invasion from the White Sea over an area today occupied by land (Shikano et al. 2010; Guo et al. 2019; this study, fig. 3). Therefore, there is a clear rationale to expect RUS-LEV to be an outlier in IBD analysis. We thus performed IBD analyses excluding the population RUS-LEV but we also report the results when including it in supplementary information 2, Supplementary Material online.

### Comparative Phylogenomic Analyses

There is evidence to suggest that the probability of genetic parallelism decreases with increasing divergence time between taxa (Conte et al. 2012; Ord and Summers 2015). To assess differences in divergence times among populations of the two species, time-calibrated phylogenetic trees were constructed using genome-wide SNPs based on the multispecies coalescent model with the program SNAPP (Bouckaert and Bryant 2012; Chifman and Kubatko 2014).

For these analyses, we selected 16 paired sampling locations from where samples of both species were available, representing all major biogeographic regions within the two species’ distribution ranges (fig. 3). SNAPP analyses were performed using filtered data sets of 12,022 SNPs for three- and 13,079 SNPs for nine-spined sticklebacks (bi-allelic SNPs > 10 kb apart, with no missing data, and posterior probability > 0.95%), following the protocols of Stange et al. (2018) and Fang et al. (2020). The time calibrations were conducted using the divergence time estimates derived from Guo et al. (2019) and Fang et al. (2020) for nine- and three-spined sticklebacks, respectively. Detailed methods of SNP filtering and phylogenetic analyses are specified in the supplementary method 7, Supplementary Material online.

#### Divergence Times and Parallel Evolution

Since genetic parallelism is expected to be a negative function of time since divergence (e.g., Conte et al. 2012), we further explored the correlation between divergence time and the level of genetic parallelism using seven freshwater population pairs from Europe (shown in supplementary fig. 6, Supplementary Material online). In each species, we first extracted the divergence times (in Ma) between pairwise intraspecific populations based on the maximum-clade-credibility summary tree, using the R-package *ape* (Paradis et al. 2004). The level of genetic parallelism for each pair of freshwater–freshwater populations was estimated by counting the proportion (relative to the entire data set) of marine–freshwater-associated LD cluster (−log_10_(*p*) > 2) loci that grouped the two freshwater populations in the PCA (individuals from both freshwater populations were found in the in-group) for a given LD cluster. Finally, the matrixes of pairwise intra-specific divergence times and levels of genetic parallelism were fitted using MLPE model similar to the IBD analyses (described above).

As processes that govern diversity levels within genomes (background selection, mutation rate, and recombination rate variation) are conserved between closely related populations (and species), different measures of diversity are correlated across the genomes of closely related populations (Dutoit et al. 2017). Here we take advantage of this correlation to detect whether outlier genomic regions have more ancient origins than neutral regions by testing whether genomic regions under selection show excess absolute divergence (*d*_XY_, Nei 1987) relative to the rest of the genome (Δ*d_XY_*) as detailed in supplementary method 8, Supplementary Material online.

## Supplementary Material

Supplementary data are available at *Molecular Biology and Evolution* online.

## Supplementary Material

msab144_Supplementary_Data
