# Supplementary material for: Population Structure Limits Parallel Evolution in Sticklebacks
Source: Mol Biol Evol. 2021 May 6;38(10):4205–21. doi: 10.1093/molbev/msab144 (PMC8476136; doi:10.1093/molbev/msab144)
Supplement: msab144_Supplementary_Data [file msab144_supplementary_data.pdf]

Content

Supplementary Methods

Supplementary Method 1 | Complexity reduction using nested three-step linkage disequilibrium network analyses (LDna).

Supplementary Method 2 | Testing associations between "synthetic alleles" from LD-clusters and ecotypes using linear mixed models.

Supplementary Method 3 | Restricted maximum likelihood (REML) analyses.

Supplementary Method 4 | Correction for multiple testing by permutation.

Supplementary Method 5 | Genotype likelihood estimation.

Supplementary Method 6 | Genetic diversity and differentiation.

Supplementary Method 7 | Comparative phylogenomic analyses.

Supplementary Method 8 | Genetic diversity and divergence in the marine–freshwater divergent genomic regions.

Supplementary Figures

Supplementary Figure 1 | Nested three-step LDna complexity reduction.

Supplementary Figure 2 | The relationship between EMMAX analyses performed on "synthetic alleles" from PCA-analyses of LD-clusters.

Supplementary Figure 3 | Cumulative numbers of outlier regions detected for randomly selected parameter/correction method combinations.

Supplementary Figure 4 | Supplementary genetic diversity estimates.

Supplementary Figure 5 | Intra-clade genetic differentiation.

Supplementary Figure 6 | The maximum-clade-credibility summary trees of the SNAPP phylogenies reported in Fig. 3, main document.

Supplementary Figure 7 | Genetic diversity and divergence in the genomic regions under selection.

Supplementary Figure 8 | Correlations between divergence time and genetic parallelism.

Supplementary Figure 9 | Sampling maps of the populations used in different analyses.

Supplementary Tables

Supplementary Table 1 | Information on LD-clusters of three- and nine-spined sticklebacks.

Supplementary Table 2 | Information on the nine-spined stickleback samples used in the study.

Supplementary Table 3 | Populations in Figure 1b, c.

Supplementary Information

Supplementary Information 1 | Methodological considerations.

Supplementary Information 2 | Outlier population (RUS-LEV) in the IBD analyses.

SUPPLEMENTARY METHODS

1 | Complexity reduction using nested three-step linkage disequilibrium network analyses (LDna)

Since allelic states of many pairs of loci can be highly correlated (in linkage disequilibrium, LD), corrections for multiple testing – assuming all loci are independent – are unnecessarily conservative. Recently, Li et al. 2018 (see also: Santure and Garant 2018) developed a method where correlated sets of loci (LD-clusters) along non-overlapping windows along chromosomes can be grouped together by LD network analyses (LDna; Kemppainen et al. 2015) and summarised by Principal Component Analyses (PCA) prior to Genome Wide Association (GWA)-analyses. Furthermore, in the presence population structuring, LD typically extends across the whole genome such that complexity reduction can be increased further by performing LDna within chromosomes or even at the whole genome level (prior to GWA-analyses). The idea is that for all (genome wide) loci that only reflect population structuring (thus cannot be reliably associated with any of the studied traits due to relatedness) only one test is sufficient, thus substantially reducing the total number of tests needed. The benefit of this, relative to window-based approaches that disregards the correlation structure among loci, is the possibility to separate different sets of correlated loci even though they are interspersed in the genome (Li et al. 2018), as demonstrated in Supplementary Fig. 1a.

Since LD-estimates between all pairs of loci from large population genomic data set (with millions of loci) cannot be considered simultaneously, we adopt a nested three-step approach to perform complexity reduction (Fang et al. 2020; Li et al. 2018) prior to downstream analyses. In LDna step 1 (LDna-1) we recursively identified LD-clusters along chromosomes where at least one locus was connected by LD>0.7 with all other loci in its cluster. These SNPs represent the whole cluster in the subsequent analysis steps (rSNPs; if several were found, one SNPs was randomly chosen; Supplementary Fig. 1a). To reduce computational speed this recursive algorithm was performed within non-overlapping windows of 1000 bi-allelic SNPs and within those windows, LD was estimated in windows of 100 SNPs with minor allele frequency, *mar*>0.05, (Fang et al. 2020). Thus, while no SNP could directly be connected to another SNP further than 100 SNPs away, they could nevertheless belong to the same cluster via links to other (intermediate) SNPs, and SNPs from different (non-overlapping) 1000 bps windows can of course become

connected in LDna-2. The median proportion of the genetic variation explained by PC1 for such LD-clusters typically exceeds 99.9%.

In the second step (LDna-2; Supplementary Fig. 1b), using the rSNPs from LDna-1, we performed LDna separately for each chromosome ignoring all singleton clusters (SNPs that were not connected to any other locus by at least LD = 0.7), as in Fang et al. 2020. In standard LDna (Kemppainen et al. 2015), all pair-wise LD values are considered simultaneously using single-linkage clustering trees, where each cluster is defined by its weakest LD-value among any pair of loci in the cluster. LD-clusters of interest are defined by two parameters; the minimum number of edges in the cluster ($|E|_{min}$, which is sensitive to both the connectedness within the cluster as well as the number of loci in a cluster) and a threshold for λ (λ_{lim}), which indicates the magnitude of how different LD-clusters are with respect to median LD when considered separately or jointly (Kemppainen et al. 2015). However, similar cluster solutions that can be obtained by different combinations of $|E|_{min}$ and λ_{lim} can also be obtained just by varying $|E|_{min}$. Since these parameters are redundant, we here considered all branches for a given $|E|_{min}$ as separate LD-clusters regardless of λ (thus removing this parameter altogether) as shown in Supplementary Fig. 1b. Since, for a given value of $|E|_{min}$, PC1 in “LD-clades” with node depths ≥0.8 (i.e. when the lowest LD value between any two loci in the cluster is ≥0.8) typically explain >>90% of the variation, these were considered as single LD-clusters; such cases increase with lower $|E|_{min}$ as this typically leads to more branching at higher LD levels. In previous implementations of the three-step LDna (Fang et al. 2020), the most interconnected SNP (the SNP with the highest median LD with all other loci in its cluster) in each LD-cluster/branch from LDna-2 was chosen as the rSNP for LDna-3. However, since the genetic variation in a cluster with many loci more efficiently and robustly can be summarised by PC-coordinates (based on all the loci in the cluster), we here opted to base the LDna-3 tree on all pairwise correlation coefficients of PC1 coordinates between the LDna-2 LD-clusters entering LDna-3 (Supplementary Fig. 1c). The matrix of all pair-wise correlation coefficients K_{PC1} were nevertheless always correlated with the corresponding matrix of LD K_{LD} based on rSNPs, so this is not likely to substantially affect the final LDna-analysis, except to make it less sensitive to the exact choice of the rSNP.

Data sets

To make a rigorous comparison, the comparative LDna analyses between three- and nine-spined sticklebacks were restricted to the Atlantic region. This is because our sampling lacked marine nine-spined stickleback populations from the Pacific regions and because three-spined sticklebacks from the Eastern Pacific region exhibit unusually high (ca. 10 times) genetic parallelism compared to other regions in the world (Fang et al. 2020).

To generate comparable datasets for three- and nine-spined sticklebacks for LDna and downstream analyses, it is important to consider that genome coverage was much higher in nine- than three-spined sticklebacks due to larger proportion of WGS samples in the former species (80.1%) than in the latter species (22.9%). Therefore, firstly, we made a list of sites across the genome for both species for which we had sufficient coverage of high-quality reads using ANGSD (-minIndDepth 1, -uniqueOnly 1, -remove_bads 1, -minMapQ 20, -minQ 20, -minInd 25%), resulting in 101,598,319 and 129,356,224 sites in three- and nine-spined stickleback genomes, respectively. Then we retained variable sites using the -SNP_pval flag in ANGSD (-SNP_pval $1e^{-6}$) and filtered by minor allele frequency (maf ≥ 0.05) using custom R scripts based on the output 'GENO' file. After this, 882,125 SNPs and 1,725,617 SNPs were retained from three- and nine-spined sticklebacks, respectively. Next we computed the number of SNPs that we would have discovered in nine-spined sticklebacks if we had sequenced the same number of bases as for the three-spined sticklebacks: $SNPs_{9s}=SNPs_{3s}/L_{3s}*L_{9s}$, where $SNPs_{9s}$ is the number of SNPs to retain in the nine-spined sticklebacks dataset, $SNPs_{3s}$ is the total number of SNPs passing quality filters in the three-spined sticklebacks dataset, and L_{3s} and L_{9s} are the total number of sequenced sites passing quality control in the three and nine-spined sticklebacks datasets, respectively. This resulted in 1,355,325 SNPs in the nine-spined stickleback dataset (101,598,319/129,356,224*1,725,617), which we subsampled from the 1,725,617 SNPs that passed quality filtering. Ultimately, 882,125 SNPs for the three- and 1,355,325 SNPs for the nine-spined stickleback (in form of genotype likelihoods) were used in the downstream comparative LDna analyses.

2 | Testing associations between "synthetic alleles" from LD-clusters and ecotypes using linear mixed models

Since population demographic events such as range expansions, bottlenecks and population growth greatly increase the variance of test statistics from “Genome-scan” based outlier methods, such methods are prone to inflated rates of false positives as well as lack power when background differentiation due to population structuring is high (Galloway et al. 2020; Hoban et al. 2016; Matthey-Doret and Whitlock 2019). Genetic regions underlying variation in locally adapted traits can be identified directly by linkage mapping or genome-wide association (GWA) approaches (e.g. Robinson et al. 2014; Savolainen et al. 2013; Vinkhuyzen et al. 2013). When studying local adaptation, however, both genome-scans and GWA-analyses estimate the same statistical association, namely that between locally adapted phenotypes and genotypes. Since all locally adapted traits are by definition in some way correlated with habitat, the expectation is that all locally adapted traits deviate from the null distribution in GWA-analyses, even if only one of them is tested (LD caused by local adaptation/parallel evolution; Kemppainen et al. 2015). Conversely, if ecotype designation/habitat itself is treated as a binary trait, all genomic regions correlated with local adaptation are expected to deviate from the null distribution. While relatedness in GWA-studies at any level of the population hierarchy (from families to populations) can also lead to false statistical associations between loci and traits of interest (i.e. *p*-value inflation; Kang et al. 2010; Kang et al. 2008), this has elegantly been solved by linear mixed model (LMM) analyses that successfully correct for the genetic relatedness in the data (Kang et al. 2008). When phenotypic values are spatially auto correlated (i.e. certain phenotypic values are mainly shared among related groups of individuals) this can, however, substantially decrease the power in GWA-studies (Kang et al. 2008). The converse can also be true; when two independently colonized and genetically divergent populations share the same heritable phenotypes, correcting for relatedness could potentially increase power to identify the associated loci (Kang et al. 2008). The main benefit of using GWA analyses to find loci associated with local adaptation is thus the ability to use a linear mixed model analysis framework adopted from GWA-studies to monitor and control for relatedness and other co-factors. Indeed, in a related field, Kemppainen et al. 2017 showed that in artificial selection experiments allele frequency change before and after selection also are prone to false positives due to relatedness, and that this could be solved by LMM accounting for relatedness by analysing survival as a binary trait (e.g., “1” for individuals remaining in the population after selection and “0” for those individuals that died). In Kemppainen et al. 2017, the correlation between

-log10(P)-values from LMM analyses and those attained from a null-distribution generated by permutation was close to unity.

Previously, the Euclidean distance between group centroids (along the two first PC-axes and adjusting for the Euclidean distances within groups), known as the cluster separation score *CSS* has been applied to detect genomic regions associated with marine-freshwater parallelism in three-spined sticklebacks (Jones et al. 2012; Kingman et al. 2020). The significance of *CSS* can be tested by permutation and it correlates with F_{ST}, but with higher resolution when differentiation is strong (Jones et al. 2012). Since PC1-coordinates from the LD-clusters typically explain >>90% of the total variation, the *CSS* in this study will be simplified to the difference in means of the PC1-coordinates between marine and freshwater individuals. This simplification, however, is likely to lead to significant loss of information when a window-based approach to *CSS* is applied, i.e. disregarding correlation structure between SNPs, as in e.g. Jones et al. 2012 and Kingman et al. 2020, and is thus only applicable in combination with complexity reduction using e.g. LDna.

Similarly to Kemppainen et al. 2017, where the significance of allele frequency differences between groups of individuals was tested for each bi-allelic SNP separately, we here show that the correlation between -log10(P)-values from permutation (performed as in Jones et al. 2012 and Kingman et al. 2020) and from LMM-analyses (treating ecotype as a binary trait, but not correcting for any other potentially confounding factors) also is close to unity (Supplementary Fig. 2). Thus, PC1 coordinates from the LD-clusters can be viewed as "synthetic multi-locus alleles" (SMLAs). Here, we thus adopt a similar approach to Kemppainen et al. 2017 to test for asssociations between the SMLA and ecotype using LMM, which allowed us to correct for potential p-value inflation due to relatedness and other potentially confounding factors. Note that in contrast to the *CSS* score used in Jones et al. 2012 and Kingman et al. 2020, our simplified *CSS* scores – that can be replaced by LMM analyses treating ecotype as binary trait – does not account for within group variances. However, the correlation between -log10(P)-values from our simplified *CSS* and the *CSS* used in Jones et al. 2012 and Kingman et al. 2020 is high (r^2=0.97, n=343; this test was performed on all nine-spined stickleback LDna-1/LDna-2 clusters with parameter settings $|E|_{min}$=20m, SNP_{min}=20, with 10^5 permutation replicates). Similarly, based on all LDna-1 clusters from the three-spined sticklebacks in this study, the linear correlation between -log10(P) from LMM and their 95% quantile F_{ST} (based on the loci from each cluster) was r^2=0.92 (n=4412). Thus, when not accounting for any confounding factors,

testing for the association between SMLAs and ecotype using LMM can replace both *CSS* (as performed in Jones et al. 2012 and Kingman et al. 2020) and F_{ST}.

3 | Restricted maximum likelihood (REML) analyses

Linear mixed models can be used to control for hidden population and/or family structure in the data by including the relatedness among individuals as a random effect. Restricted maximum likelihood (REML)-based approaches have been widely used to evaluate the regression parameters and variance components in such models (Kang et al. 2010; Kang et al. 2008). EMMAX (Kang et al. 2010) estimates the variance components once based on an intercept model and subsequently fixes them to evaluate the effect and statistical significance of the SNPs. This approach is preferred over its predecessor due to its computational efficiency while maintaining both power and the ability to control for false positives (Kang et al. 2010; Kang et al. 2008). Association tests here are based on the coordinates of the first PC extracted from PCA (i.e. the SMLAs) on all loci from a given LD-cluster (Li et al. 2018; Fig. 1c) defined as:

$$y_i = \theta_0 + \sum_{l=1}^{m_k} W_{il}\theta_l + u_i + \varepsilon_i, \varepsilon_i \sim N\left(0, \sigma_e^2\right)$$

where $W_{il}(l = 1, \dots, m_k)$ represents the PC coordinates in *k*th LD-cluster; where M is the total number of clusters), θ_0 is the intercept, $W_{il}(l=1,\dots,m_k)$ is the regression parameter of the given PC and u_i is the random effect defined as $u = [u_i \dots, u_n] \sim MVN(0, \sigma_g^2 A)$ with known n x n sized relationship (kinship) matrix **A** and unknown variance σ_g^2 (Li et al. 2018). Given uncertainties in low coverage whole genome sequence data, we obtained the relationship matrix **A** (based on all available loci) in a probabilistic framework from individual allele frequencies estimated iteratively based on inferred population structure from genotype likelihoods using the software *PCAngsd* (Korneliussen et al. 2014).

4 | Correction for multiple testing by permutation

Although computationally much more intensive, an alternative but potentially less conservative alternative to the “false discovery rate” method to control the family-wise error (FWER) is by permutation that accounts for the correlation structure among the multiple tests (Efron 2010; Joo et al. 2016; Li et al. 2018). This method is particularly useful in combination with LD-based complexity reduction, where the total number of tests can in many cases substantially be lowered, as demonstrated by Li et al. 2018. To correctly account for the among individual correlation structure in the data we generated phenotypic data under the null-hypothesis by drawing phenotypic data from a multivariate normal distribution θ_0 and then using EMMAX to calculate the test-statistics on each sample. The resulting empirical distributions of the test statistics were then used to adjust p-values to control the multiplicity as in Li et al. 2018. Since we are testing for associations between loci and ecotype (treated as a binary trait), we treated the phenotypes as threshold traits, with the number of freshwater and marine ecotypes equal to that observed in the real data.

5 | Genotype likelihood estimation

In the step of quality filtering in ANGSD, we retained only bases with a minimum Phred score of 20 and a minimum mapping quality of 25 (-minQ 20 and -minMapQ 25). We retained sites with less than 20% missing data and a minimum read depth of two for each individual following Fang et al. 2020 to account for low sequence-depth data and computed genotype likelihoods for variants that had a *p*-value smaller than 1e-6 (-SNP_pval 1e-6). The sex chromosomes were excluded (chromosome 19 [group XIX] in the three-spined stickleback; Kitano et al. 2009; Natri et al. 2013; chromosome 12 [LG 12] in the nine-spined stickleback; Natri et al. 2019; Rastas et al. 2015; Shapiro et al. 2009). The full scripts of ANGSD pipelines can be found from Fang et al. 2020 (https://doi.org/10.5061/dryad.b2rbnzsb1).

6 | Genetic diversity and differentiation

For π and θ, site allele frequency likelihoods and the site frequency spectrum (SFS) were estimated for each population in ANGSD. The summary statistics of π and θ for each population were calculated using a custom R scripts from Momigliano et al. 2021. Scripts

can be found in: https://github.com/Nopaoli/Demographic-Modelling/blob/master/Diversity_fromSFS/Stats_from_SFS_TD.R. To calculate *H*, we first estimated the SFS for each individual and divided the number of segregating sites (the second entry of the SFS) by the sum of the SFS. Population estimates of π and θ were only applied to the populations consisting of multiple individuals (sample size n ≥ 2). The samples from Jones et al. 2012 in the dataset of three-spined sticklebacks (c.f. Fang et al. 2020) were excluded in the *H* calculation due to low read depth (≤ 1). The Supplementary Fig. 9 presents the sampling maps of the populations used in different analyses.

Since some sampled populations are known to be admixed (Feng et al. 2020; Guo et al. 2019), their genetic diversity was expected to be elevated. Therefore, the analyses of *H*, π and θ were conducted both excluding and including the known admixed nine-spined stickleback populations (information from Guo et al. 2019 and Feng et al. 2020), and both results were reported (Fig. 1 and Supplementary Fig. 4). Specifically, seven out of 29 freshwater populations (BEL-MAL, DEN-RES, PP-FR-MAR, PP-RU-BLS, PP-RU-GOR, PP-RU-KVA, PP-RU-MAG) were identified to be admixed with other *Pungitius* species (Guo et al. 2019), and six out of seven marine populations (GER-RUE, FIN-HEL, SWE-BOL, FIN-KIV, DEN-NOR, SWE-FIS) have experienced varying degree of introgression between divergent *P. pungitius* lineages (Feng et al. 2020).

To calculate F_{ST} in each species, genotypes were called in ANGSD (-doVcf 1) with additional filtering on the genotype posterior probability (-postCutoff 0.95). VCFtools v.0.1.15 (Danecek et al. 2011) was used for the downstream SNP filtering, setting maximum missing genotypes to 20% (--max-missing 0.8), a minimum minor allele frequency to 0.05 (--maf 0.05) and minimum distance between any two sites to 30,000 bp (--thin 30,000). We then used this subset of high quality, called genotypes to estimate global F_{ST} for each ecotype and the pairwise F_{ST} between all populations with the R packages *hierfstat* (Goudet 2005) and *StAMPP* (Pembleton et al. 2013), respectively.

7 | Comparative phylogenomic analyses

For both species we filtered SNPs called from ANGSD in VCFtools, retaining only “unlinked” (i.e. no closer than 10 kb; --thin 10000) bi-allelic loci (--min-alleles 2; --max-alleles 2) with no missing data (--max-missing 1) and a minor allele frequency above 0.01.

For nine-spined sticklebacks, the first calibration was the time to the most recent common ancestor (TMRCA) of all lineages, 2.69 Mya (million years ago, 95% Highest Posterior Density [HPD] interval: 1.87–3.57 Mya). The second was the TMRCA of European lineages at 0.67 Mya (95% HPD interval: 0.43–0.92 Mya). For three-spined sticklebacks, the first calibration was the TMRCA of all lineages, 191.7 Kya (thousands of years ago; mean of the time estimates in Fang et al. 2020b [36.9–346.5 Kya]). The second was the divergence time between the Pacific and Atlantic lineages, 128.1 Kya (29.5–226.6 Kya). The third one was the TMRCA of the Northern European lineages, 53.25 Kya (11.3–95.2 Kya). The calibration points are visualized in the Supplementary Fig. 6.

To perform SNAPP analyses, the input XML file was prepared using a Ruby script “snapp_prep.rb” (github.com/mmatschiner/snapp_prep). Stationarity and convergence of 100 million Markov-Chains Monte Carlo (MCMC) from SNAPP were checked in TRACER v1.6 (ESS > 1000; beast.bio.ed.ac.uk/tracer/). A maximum-clade-credibility summary tree was generated using TreeAnnotator (Drummond et al. 2012). The SNAPP trees were visualised by the program DensiTree v.2.2.6 (Bouckaert 2010) with a 10% burn-in for each MCMC chain.

8 | Genetic diversity and divergence in the marine–freshwater divergent genomic regions

To see whether genomic regions under selection have more ancient origins than neutral regions, we tested whether the absolute genetic divergence (d_{XY}, Nei 1987) in the genomic regions under selection exceeds that in the rest of the genome. All freshwater populations considered are the results of postglacial invasions, and hence mean d_{XY} between freshwater and geographically close marine populations is expected to be low, i.e. to approximate π from the ancestral population (see discussion in Ravinet et al. 2017). If selection is acting on a novel or rare variant, we can expect a high peak in F_{ST} but not an increase in d_{XY}, as allelic differentiation would be a result of reduced π in the population

experiencing selection (i.e. a selective sweep; Cruickshank and Hahn 2014). In contrast, if selection is acting on ancient haplotypes, a peak in F_{ST} is likely to be driven by an increase in d_{XY}, rather than a decrease in π (e.g. Nelson and Cresko 2018). Comparing d_{XY} in regions underlying parallel evolution with the rest of the genome could provide information on the evolutionary origin of the SGV under selection, but such comparison is not straightforward. Like other measures of diversity (π and θ), d_{XY} is strongly affected by the heterogeneity in background selection, recombination rates and mutation rates across the genome (Charlesworth et al. 1993). Since the processes that govern diversity levels within genomes (background selection, mutation rate and recombination rate variation) are conserved between closely related populations (and species), different measures of diversity (π and d_{XY}) are correlated across the genome of closely related populations. Dutoit et al. 2017 demonstrated this correlation is maintained even after lineage sorting is complete, suggesting shared ancestral variation cannot explain this pattern.

Here we take advantage of this correlation to detect whether genomic regions under selection show excess absolute divergence. We start from the assumption that marine populations within the same regions are proxies of ancestral diversity. For each genomic region involved in parallel evolution we then run a linear model where π_{marine} is the predictor of d_{XY} across the genome, including 100 random genomic regions as well as the region under selection. This yields a distribution of residuals (Δd_{XY}), where a Δd_{XY} of 0 represents the d_{XY} predicted by the ancestral diversity. This is conceptually similar to Nei's measure of relative divergence — d_{a}=d_{XY}-(π_x π_y)/2, (Nei and Li 1979) — but with the difference that we use π of the marine population as a proxy of ancestral diversity rather than the mean of π between both populations. Furthermore, the residuals are cantered so that positive and negative values represent higher and lower relative divergence than the mean across all regions. We use the random 100 neutral genomic regions to infer the confidence intervals for the neutral distribution, and then compare to Δd_{XY} of the target region. We use a similar approach to detect whether regions under selection show lower diversity in the freshwater populations than the rest of the genome (i.e. if peaks in F_{ST} are caused by selective sweeps). As for d_{XY}, we run a linear regression between $\pi_{freshwater}$ and π_{marine} (lm $\pi_{freshwater}$ ~ π_{marine}) based on the same 101 genomic regions. We refer to the residuals of the model as $\Delta\pi$. A negative $\Delta\pi$ in the genomic region under selection would

be suggestive of a reduction in π relative to the expected difference in π between the two species.

Estimates of genetic diversity (π and d_{XY}) were calculated as follows. Focusing on the Norwegian Sea freshwater three-spined sticklebacks and White & Barents Seas freshwater nine-spined sticklebacks (see Materials and Methods), we treated one focal freshwater population and one marine population across the Atlantic as a marine–freshwater population pair. Only the populations with at least two samples were considered and all population pairs were found. For each candidate genomic region in a LD-cluster (5kbp upstream plus 5kbp downstream of the loci with the highest F_{ST}), we calculated π in each ecotype and d_{XY} between the two ecotypes in each population pair. We first estimated 1D-SFS of each population and 2D-SFS of both populations using ANGSD. Then we calculated π and d_{XY} based on 1D-SFS and 2D-SFS respectively using custom scripts derived from Momigliano et al. 2021. Scripts can be found in: github.com/Nopaoli/Demographic-Modelling/tree/master/Diversity_fromSFS. Models of Δd_{XY} *and* $\Delta\pi$ were performed in R for each population pair, and the results (Δd_{XY}, $\Delta\pi$ and r^2) were averaged across all population pairs in Fig. 5 and Supplementary Fig. 7 for presentation.

SUPPLEMENTARY FIGURES

Supplementary Figure 1 | Nested three-step LDna-complexity reduction. First, (a) sets of highly correlated loci (as indicated by heatmap where light shade indicates stronger LD) along windows within chromosomes can be grouped together into LD-clusters, despite them being interspersed and overlapping along the chromosome due to “mosaic-like” LD. From each cluster one rSNP (connected to all other SNPs in its cluster by high LD) is taken to represent the whole cluster in LDna-2. Second, (b) using an LD-matrix K_{LD} between all rSNPs from (a) and single linkage clustering standard LDna is performed separately for each chromosome. Here, each branch is considered as an LD-cluster, with the size and correlation determined by $|E|_{min}$ (the minimum number of edges in the cluster; higher values yielding larger but less correlated LD-clusters). Third, (c), a correlation matrix K_{PC1} was constructed based on the squared correlation coefficients between SMLAs for all pairwise comparisons between LDna-1 clusters which is then used to construct a genome wide correlation tree. Note that in LDna-3 all LDna-2 clusters, regardless of chromosome, are included. All LD-clusters identified in LDna-1 that are not part of any LDna-2 clusters but contain $>SNP_{min}$ number of loci are also included in LDna-3 analyses. Lastly, any LDna-1/2 clusters correlated at $>Cor_{th}$ will be considered a single independent LDna-3 cluster. This step can significantly reduce the number of tests by pooling together genome wide loci that only reflect population structuring.

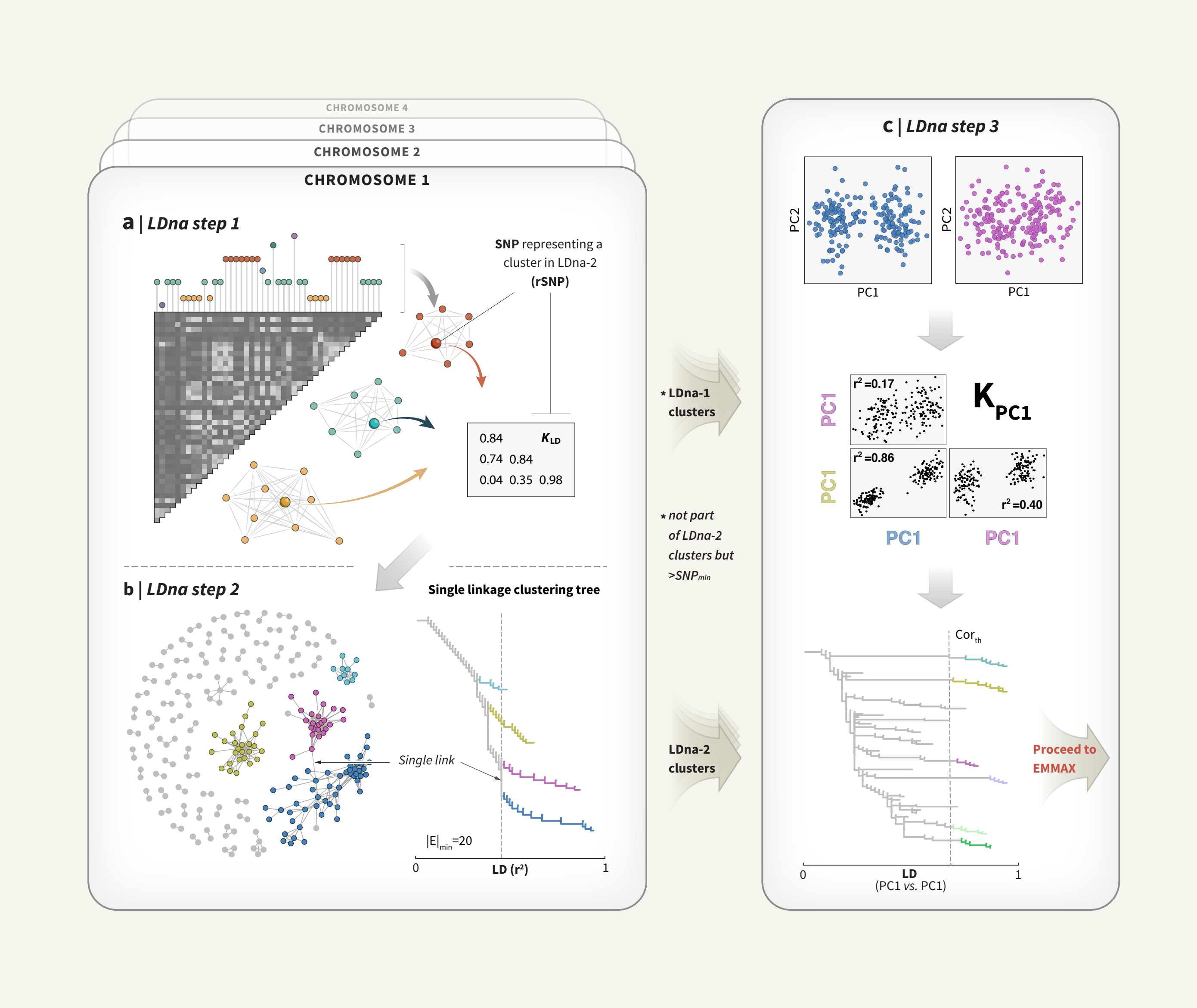

CHROMOSOME 4
CHROMOSOME 3
CHROMOSOME 2
CHROMOSOME 1
a | LDna step 1
SNP representing a cluster in LDna-2 (rSNP)
0.84 K_LD
0.74 0.84
0.04 0.35 0.98
b | LDna step 2
Single linkage clustering tree
Single link
|E|min=20
0
LD (r²)
1
* LDna-1 clusters
* not part of LDna-2 clusters but >SNPmin
LDna-2 clusters
c | LDna step 3
PC2
PC1
PC2
PC1
r² =0.17
K PC1
PC1
PC1
r² =0.86
r² =0.40
PC1
PC1
Cor th
Proceed to EMMAX
0
LD
(PC1 vs. PC1)
1

Supplementary Figure 2 | The relationship between EMMAX analyses performed on "synthetic alleles" from PCA-analyses of LD-clusters. Data points represent analyses from 1000 randomly chosen LDna-1 clusters from the three- and nine spined sticklebacks data sets testing for associations between SMLAs (PC1-coordinates from LD-clusters) and ecotype either by (y-axis) EMMAX, treating ecotype as a binary trait and not including any co-factors or random effects or by (x-axis) permutation ($n=10^5$). In both species, the relationship is close to unity, with slightly larger variance for nine-spined sticklebacks.

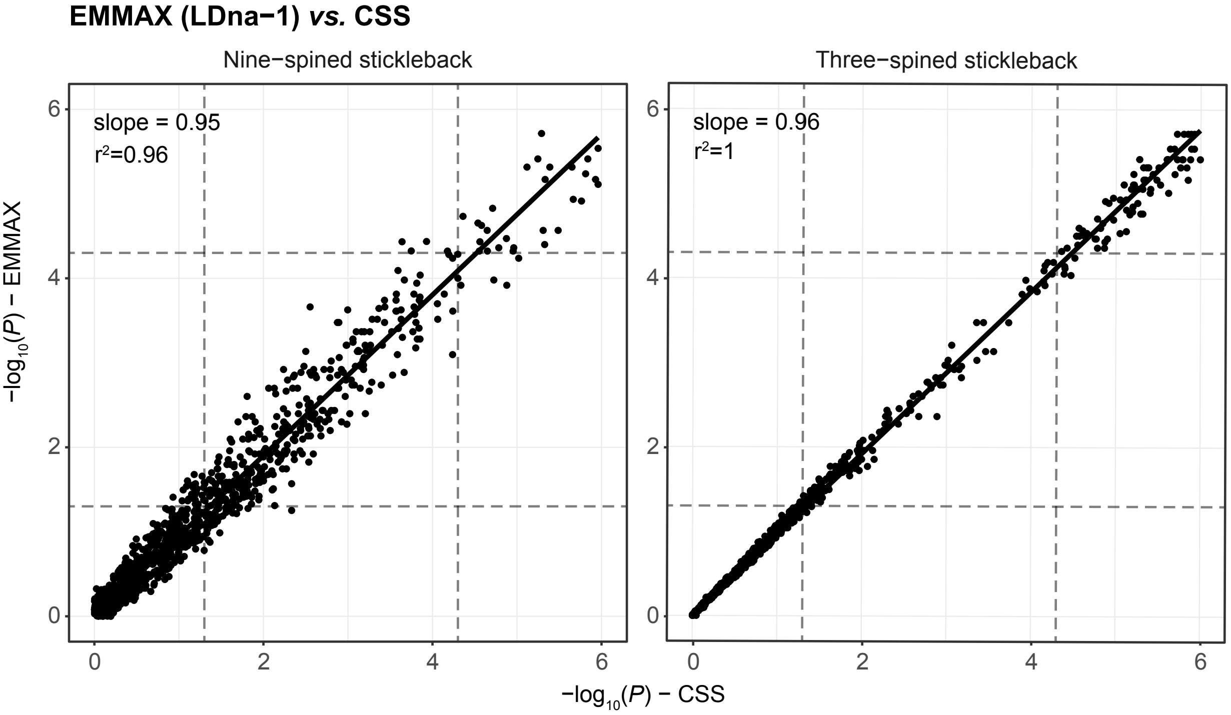

Supplementary Figure 3 | Cumulative numbers of outlier regions detected for randomly selected parameter/correction method combinations. Genomic regions associated with marine-freshwater differentiation was tested with 45 orthogonal parameter combinations of $|E|_{min}$, SNP_{min} and Cor_{th} and four methods to correct for multiple testing and p-value inflation ("A+fdr, "A+perm", "GC+fdr" and "perm", se main text for details). Black lines show the mean (n=100) cumulative increase of significant outlier regions when sequentially including more parameter/correlation method combinations (in total 144) in random order, with solid line indicating regions with $C \geq 0$, dashed line indicating regions with $C \geq 0.05$ and dotted line indicating regions with $C \geq 0.1$. In each panel from left to right the level of one parameter is fixed while other parameter combinations are randomly sampled. This shows the influence of each parameter on the number of outlier regions. For instance, the most "successful" $|E|_{min}$ settings were $|E|_{min}$=20 for three-spined but $|E|_{min}$=10 for nine-spined sticklebacks (left panel). Similarly, the most successful Cor_{th} value for three-spined sticklebacks was Cor_{th}=10, while this parameter had less influence for nine-spined sticklebacks (right panel). Both reflect the fact that outlier regions affect a larger number of correlated loci in three- compared to nine-spined sticklebacks and that a given parameter setting can be more successful (in detecting significant outlier regions) in one species compared to another.

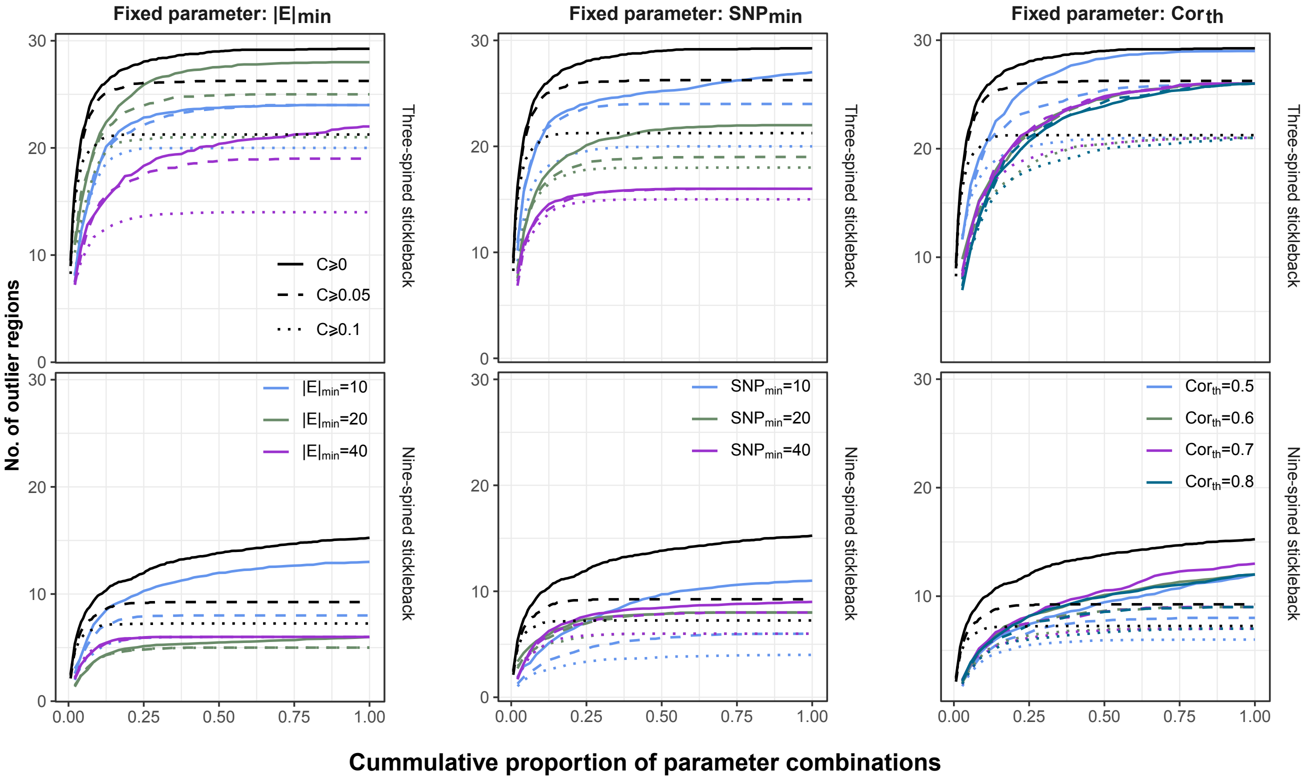

Supplementary Figure 4 | Supplementary genetic diversity estimates. Genetic diversity between the two species: (a, b) Watterson's theta (θ), (c) nucleotide diversity (π) and (d) heterozygosity (*H*). (b-d) Genetic diversity incorporating the known hybridised nine-spined stickleback populations in analyses (see Materials and methods). (e) The *H* between admixed populations and non-admixed populations of nine-spined sticklebacks. Statistics are reported in the results.

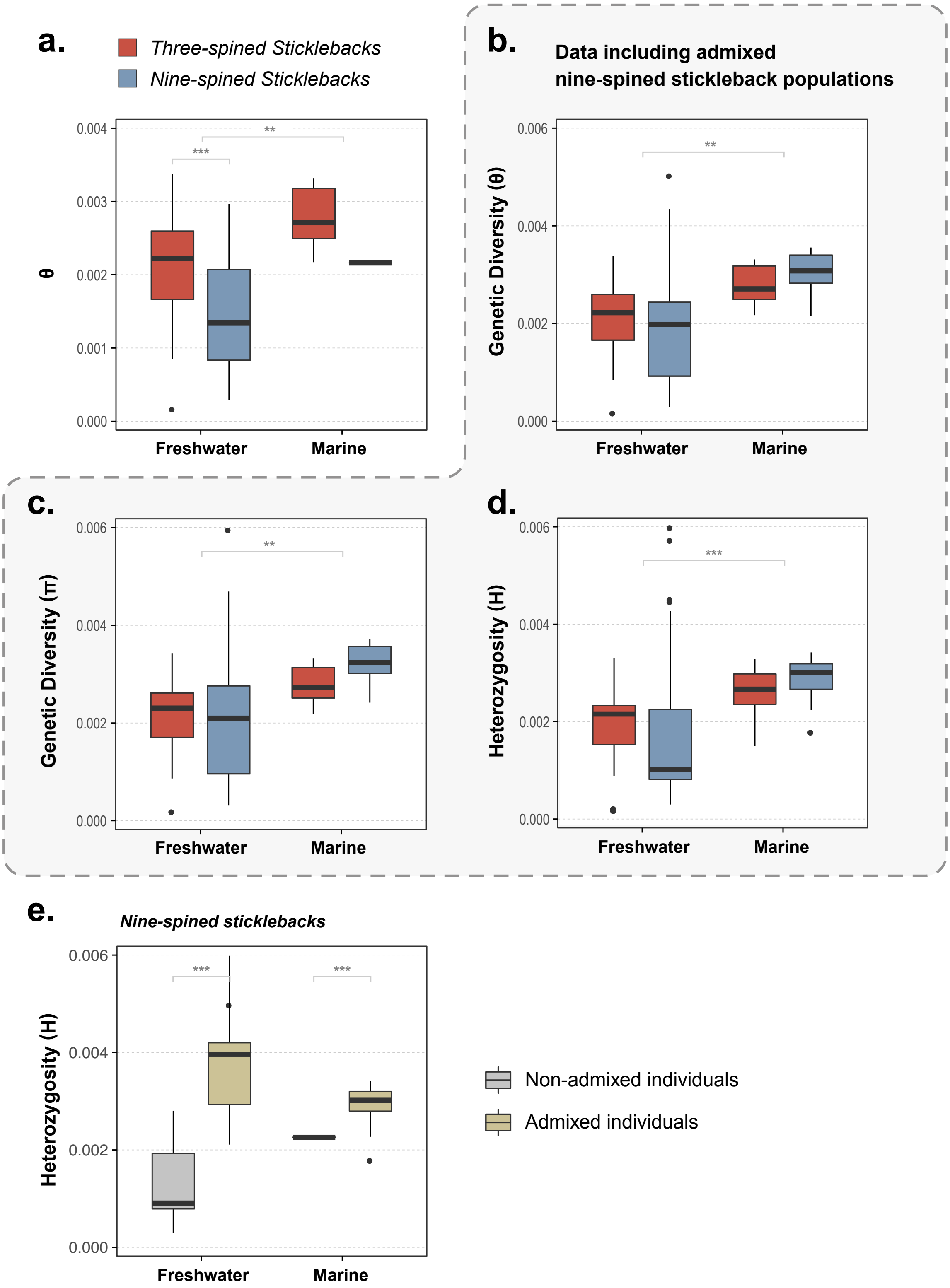

Supplementary Figure 5 | Intra-clade genetic differentiation. Boxplots of pairwise F_{ST} between populations within genetic clades in nine-spined sticklebacks compared to three-spined sticklebacks. Genetic differentiation is higher in nine-spined sticklebacks whether we consider freshwater-freshwater, marine-freshwater or marine-marine comparisons. Note that data for all ecotype contrasts were not available for all clades.

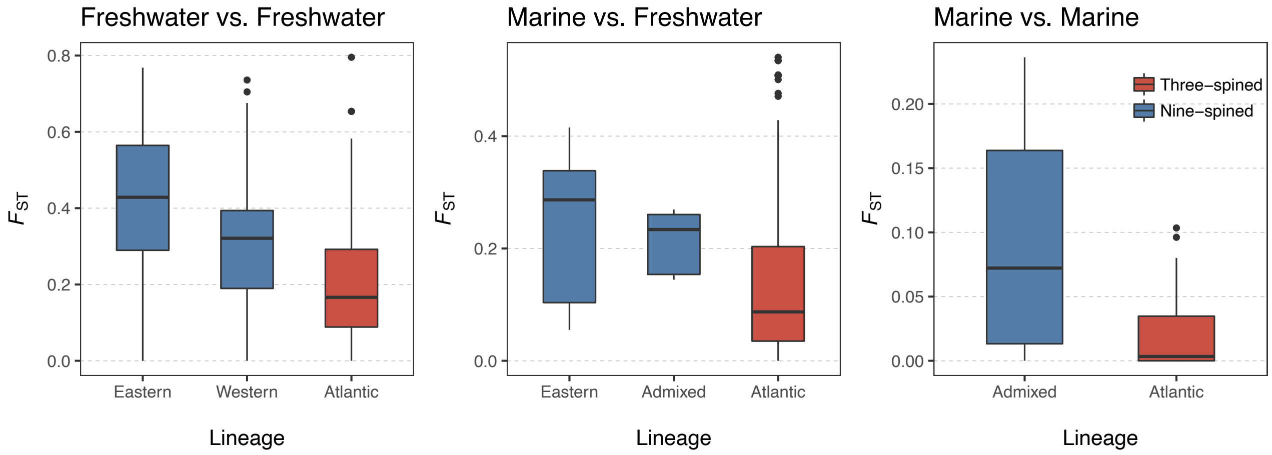

Supplementary Figure 6 | The maximum-clade-credibility summary trees of the SNAPP phylogenies reported in Fig. 3. (a) The tree of nine-spined sticklebacks. (b) The tree of three-spined sticklebacks. The numbers in the nodes represent divergence time estimates in form of “mean divergence age (Mya) [range of 95% highest posterior density (HPD) intervals]”. The circles (A, B and C) indicate the calibration points applied in the SNAPP analyses. The populations in red rectangles are used to analyse the correlation between divergence times and parallelism (see Materials and methods for details and population abbreviations).

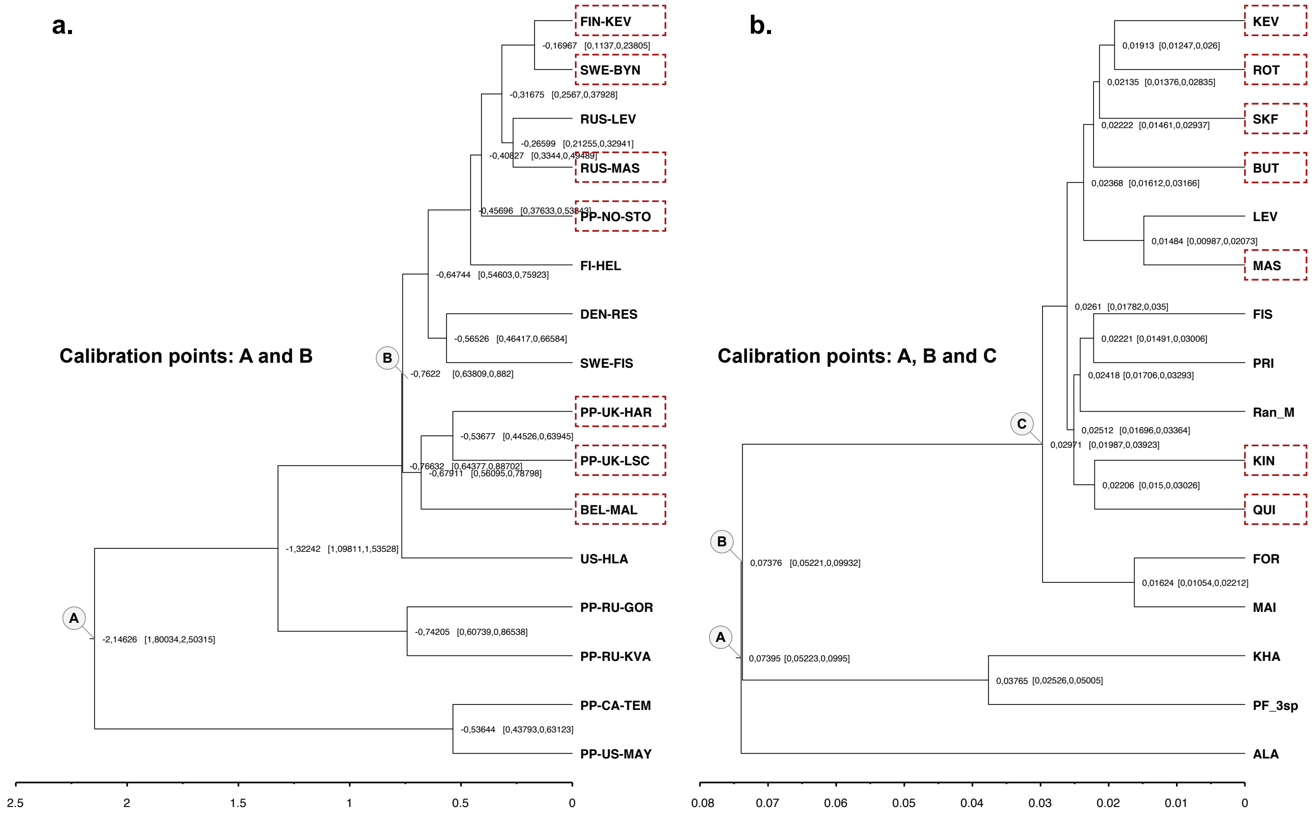

Supplementary Figure 7 | Genetic diversity and divergence in the genomic regions under selection. Data for three- (No. 1-26) and nine-spined sticklebacks (No. 27-35) are presented. In each panel, the upper plot indicates the position of the candidate region with red arrow. The lower plot summarises the residuals of linear regression models based on the genetic diversity ($\Delta\pi$) and genetic divergence (Δd_{XY}) derived from marine–freshwater population pairs in corresponding LD-cluster (see Materials and methods). The correlation coefficient (R^2) is shown as an averaged value across all models from different population pairs. All models are statistically significant (P<0.001).

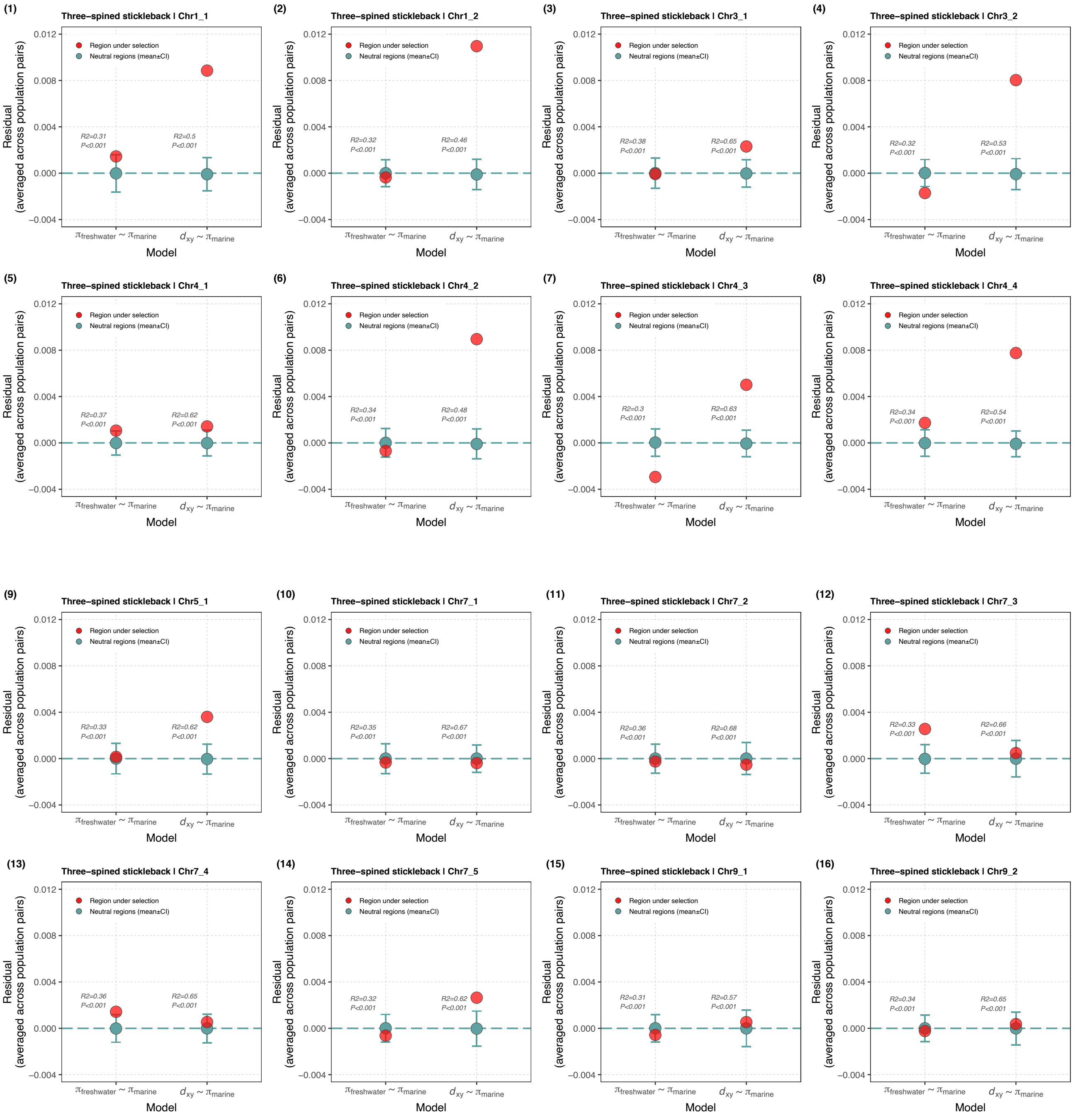

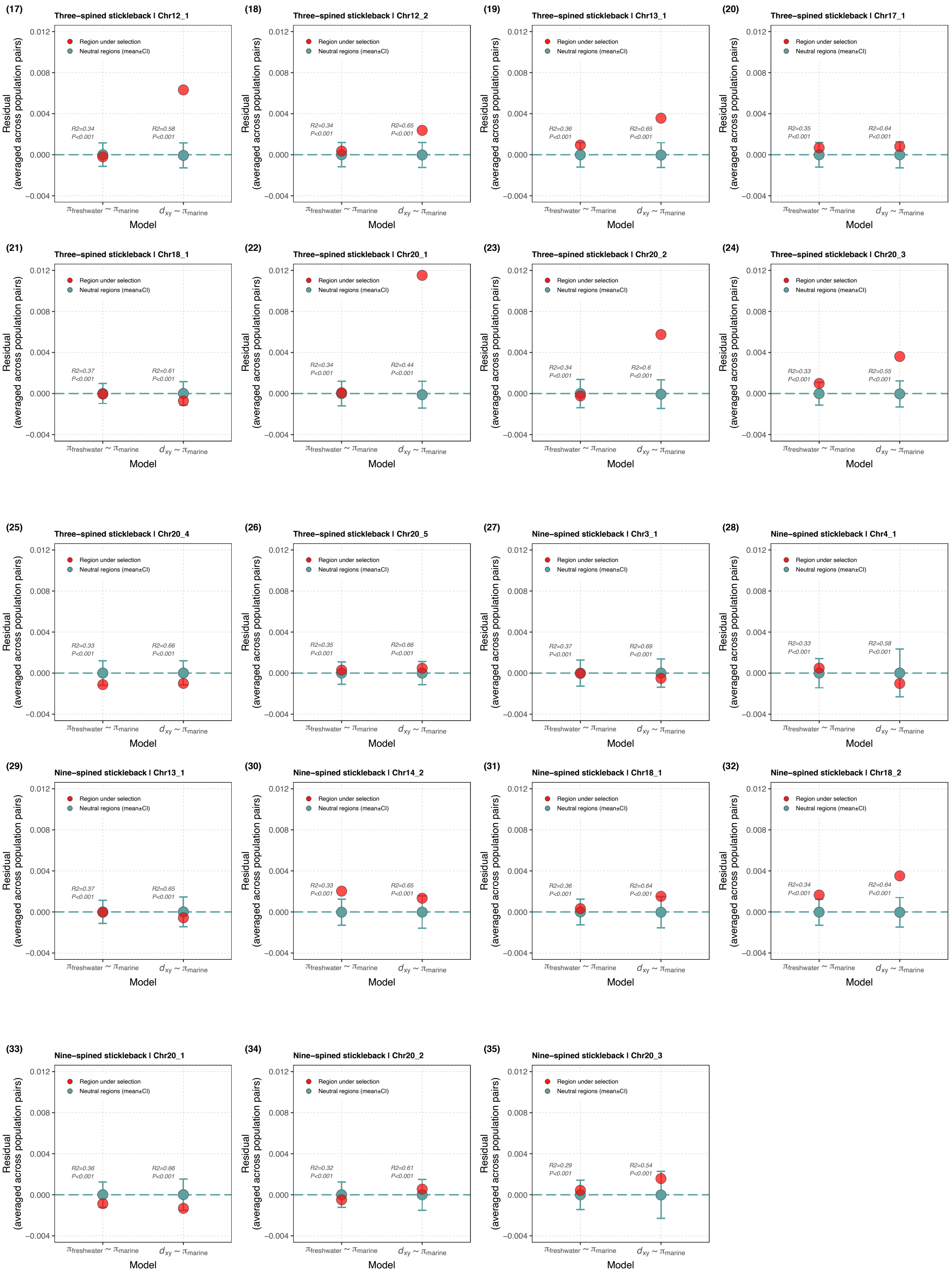
(17) Three−spined stickleback | Chr12_1
(18) Three−spined stickleback | Chr12_2
(19) Three−spined stickleback | Chr13_1
(20) Three−spined stickleback | Chr17_1
(21) Three−spined stickleback | Chr18_1
(22) Three−spined stickleback | Chr20_1
(23) Three−spined stickleback | Chr20_2
(24) Three−spined stickleback | Chr20_3
(25) Three−spined stickleback | Chr20_4
(26) Three−spined stickleback | Chr20_5
(27) Nine−spined stickleback | Chr3_1
(28) Nine−spined stickleback | Chr4_1
(29) Nine−spined stickleback | Chr13_1
(30) Nine−spined stickleback | Chr14_2
(31) Nine−spined stickleback | Chr18_1
(32) Nine−spined stickleback | Chr18_2
(33) Nine−spined stickleback | Chr20_1
(34) Nine−spined stickleback | Chr20_2
(35) Nine−spined stickleback | Chr20_3
Region under selection
Neutral regions (mean±CI)
Residual (averaged across population pairs)
Model
$\pi_{freshwater} \sim \pi_{marine}$
$d_{xy} \sim \pi_{marine}$

Supplementary Figure 8 | Correlations between divergence time and genetic parallelism. The correlation between divergence time and genetic parallelism was tested in (a) three- and (b) nine-spined stickleback population pairs, respectively. Seven pairs of populations shown in the phylogenies in Fig. 3 were used in these analyses. Specific populations are presented in the Supplementary Fig. 6. Each dot represents a pairwise comparison. The x-axis presents the divergence time (Mya) of the populations in each pair, while the y-axis displays the proportion of loci involved showing genetic parallelism in each population pair. The correlation between divergence times and genetic parallelism was tested using maximum-likelihood population-effects (MLPE) model.

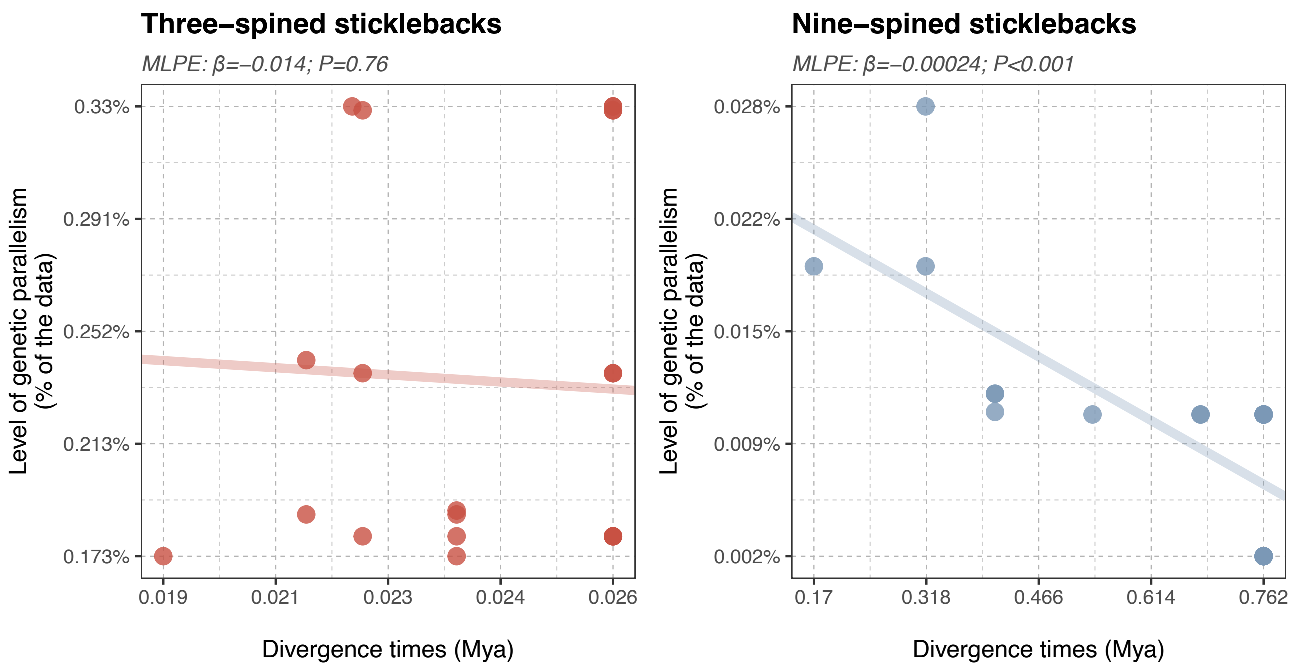

Supplementary Figure 9 | Sampling maps of the populations used in different analyses. (a) All populations in the study. (b) Populations used in the analyses of individual heterozygosity (*H*). (c) Populations used in the analyses of differentiation (F_{ST}), nucleotide diversity (π) and Watterson's theta (θ). (d) European populations used in IBD analyses. (e) Populations used in SNAPP analyses. Population identities in Fig. 3 are given on the bottom-right. See Method for justifications for the sampling in different analyses.

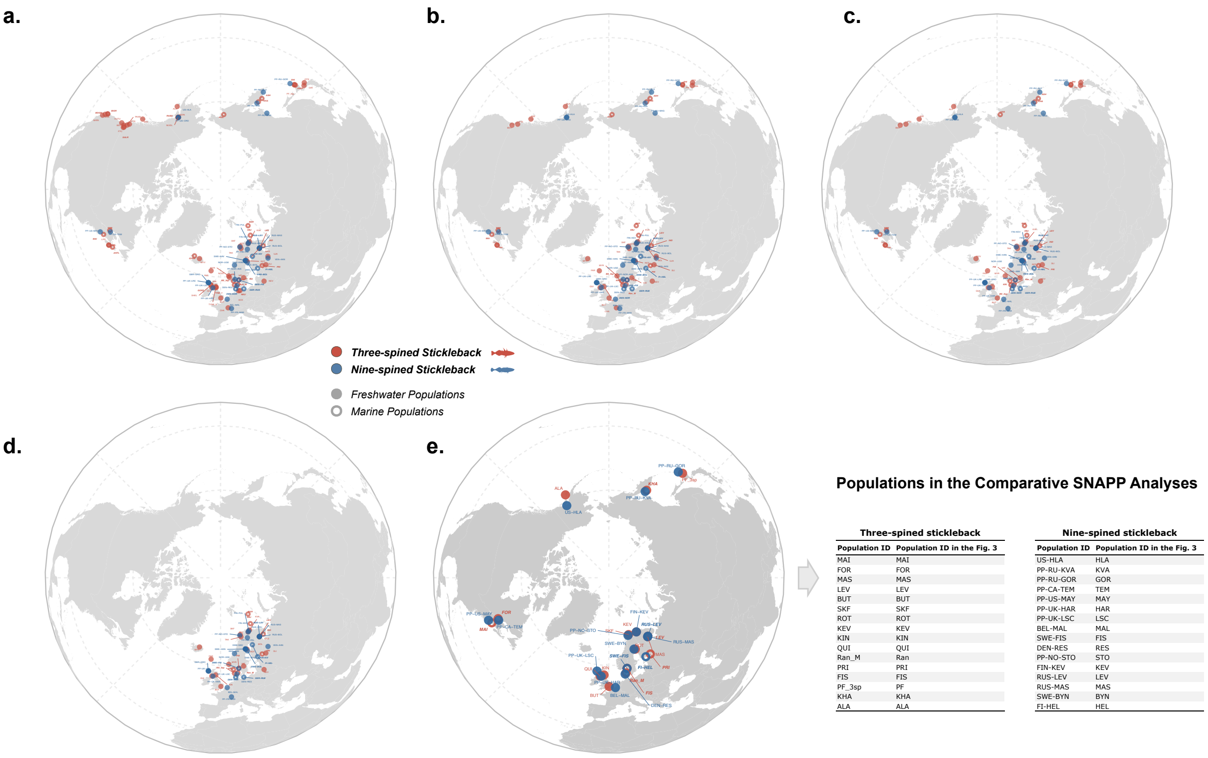

Populations in the Comparative SNAPP Analyses

Three-spined stickleback

Population ID	Population ID in the Fig. 3
MAI	MAI
FOR	FOR
MAS	MAS
LEV	LEV
BUT	BUT
SKF	SKF
ROT	ROT
KEV	KEV
KIN	KIN
QUI	QUI
Ran_M	Ran
PRI	PRI
FIS	FIS
PF_3sp	PF
KHA	KHA
ALA	ALA

Nine-spined stickleback

Population ID	Population ID in the Fig. 3
US-HLA	HLA
PP-RU-KVA	KVA
PP-RU-GOR	GOR
PP-CA-TEM	TEM
PP-US-MAY	MAY
PP-UK-HAR	HAR
PP-UK-LSC	LSC
BEL-MAL	MAL
SWE-FIS	FIS
DEN-RES	RES
PP-NO-STO	STO
FIN-KEV	KEV
RUS-LEV	LEV
RUS-MAS	MAS
SWE-BYN	BYN
FI-HEL	HEL

SUPPLEMENTARY TABLES

Supplementary Table 1 | Information on LD-clusters of three- and nine-spined sticklebacks.

Regional EMMAX analyses of the association between "synthetic multi-locus alleles" and ecotype based on loci from significant LD-clusters for a given outlier genomic region (after corrections for p-value inflation or permutation)

Sp	Chr	Pos	Region	No. loci	Fst (Q95%)	C	Range	P_min	cor_A|WB_Sea	cor_unrl|WB_Sea	cor_A|Baltic	cor_unrl|Baltic	cor_A|NorthSea	cor_unrl|NorthSea	cor_A|Norwegian	cor_unrl|Norwegian	P_A|WB_Sea	P_unrl|WB_Sea	P_A_GC|WB_Sea	P_unrl_GC|WB_Sea	P_A|Baltic	P_unrl|Baltic	P_A_GC|Baltic	P_unrl_GC|Baltic	P_A|NorthSea	P_unrl|NorthSea	P_A_GC|NorthSea	P_unrl_GC|NorthSea	P_A|Norwegian	P_unrl|Norwegian	P_A_GC|Norwegian	P_unrl_GC|Norwegian
3sp	Chr1	23981492	Chr1_2	1341	0.856	0.958	650035	1.00E-04	0.243	0.486	0.39	0.182	0.78	0.754	0.544	0.856	0.552	0	0.176	0.007	0.077	0.857	0.041	0.451	0	0	0	0	0.001	0	0.002	0
3sp	Chr5	109173367	Chr5_1	50	0.835	0.896	18569	1.00E-04	0.261	0.437	0.454	0.654	0.402	0.237	0.531	0.802	0.462	0.003	0.144	0.017	0.01	0	0.015	0	0.031	0.418	0.03	0.293	0.001	0	0.002	0
3sp	Chr12	257876940	Chr12_1	77	0.558	0.701	1.54E+05	1.00E-04	0.148	0	0.212	0.257	0.405	0.437	0.305	0.638	0.934	0.999	0.428	0.96	0.817	0.544	0.304	0.258	0.031	0.017	0.029	0.032	0.19	0	0.111	0
3sp	Chr20	366142830	Chr20_1	183	0.835	0.625	8.22E+04	1.00E-04	0.118	0.219	0.492	0.601	0.399	0.307	0.61	0.86	0.964	0.535	0.543	0.283	0.003	0	0.007	0.002	0.036	0.176	0.032	0.159	0	0	0	0
3sp	Chr4	87044465	Chr4_2	135	0.760	0.583	4.23E+04	3.00E-04	0.055	0.032	0.373	0.386	0.577	0.504	0.613	0.878	0.978	0.999	0.792	0.918	0.097	0.065	0.053	0.071	0	0.002	0.001	0.011	0	0	0	0
3sp	Chr12	260204121	Chr12_2	52	0.858	0.562	25943	1.00E-04	0.077	0.295	0.374	0.453	0.442	0.313	0.477	0.754	0.973	0.166	0.707	0.132	0.1	0.015	0.051	0.028	0.015	0.171	0.016	0.148	0.004	0	0.007	0
3sp	Chr7	149929482	Chr7_4	252	0.342	0.542	1.39E+06	1.00E-04	0.118	0.276	0.263	0.335	0.385	0.363	0.319	0.658	0.97	0.235	0.537	0.164	0.555	0.162	0.194	0.127	0.057	0.077	0.04	0.085	0.164	0	0.094	0
3sp	Chr20	374832771	Chr20_3	59	0.857	0.444	1.60E+04	1.00E-04	0.274	0.454	0.327	0.443	0.327	0.212	0.522	0.811	0.387	0	0.122	0.013	0.228	0.018	0.095	0.033	0.146	0.413	0.088	0.358	0.001	0	0.003	0
3sp	Chr13	282190862	Chr13_1	52	0.363	0.417	2.33E+04	1.00E-04	0.155	0.352	0.13	0.361	0.308	0.493	0.473	0.792	0.94	0.055	0.406	0.066	0.974	0.107	0.547	0.096	0.193	0.004	0.11	0.013	0.004	0	0.008	0
3sp	Chr4	94084384	Chr4_3	255	0.565	0.389	99349	3.00E-04	0.155	0.055	0.164	0.245	0.367	0.245	0.585	0.867	0.935	0.988	0.411	0.827	0.949	0.596	0.435	0.286	0.08	0.414	0.051	0.275	0.001	0	0.001	0
3sp	Chr7	153052832	Chr7_5	97	0.722	0.361	3.83E+04	1.00E-04	0.084	0.179	0.161	0.105	0.318	0.519	0.465	0.78	0.996	0.759	0.683	0.395	0.941	0.908	0.447	0.682	0.172	0.002	0.099	0.008	0.004	0	0.01	0
3sp	Chr3	58199692	Chr3_2	36	0.791	0.333	13749	1.00E-04	0.184	0.351	0.202	0.152	0.339	0.182	0.631	0.887	0.878	0.058	0.322	0.067	0.853	0.873	0.33	0.537	0.14	0.498	0.075	0.439	0	0	0	0
3sp	Chr4	94099576	Chr4_4	127	0.531	0.229	54496	4.00E-04	0	0.055	0.219	0.396	0.439	0.469	0.354	0.695	0.954	0.998	0.971	0.806	0.801	0.051	0.286	0.062	0.015	0.008	0.016	0.019	0.08	0	0.059	0
3sp	Chr17	342328537	Chr17_1	11	0.780	0.194	3.61E+02	1.00E-04	0.161	0.351	0.13	0.342	0.335	0.341	0.205	0.452	0.947	0.057	0.388	0.067	0.961	0.148	0.552	0.117	0.139	0.121	0.08	0.111	0.572	0.005	0.305	0.024
3sp	Chr20	382155619	Chr20_4	11	0.443	0.194	3.40E+05	1.00E-04	0.077	0.1	0	0.063	0.182	0.281	0.19	0.438	0.991	0.955	0.691	0.659	0.997	0.96	0.926	0.814	0.337	0.251	0.374	0.202	0.553	0.005	0.349	0.03
3sp	Chr20	382155619	Chr20_5	12	0.298	0.194	4.16E+05	1.00E-04	0.122	0.378	0.114	0.114	0.335	0.425	0.045	0.214	0.968	0.032	0.523	0.045	0.952	0.927	0.608	0.65	0.132	0.017	0.08	0.038	0.782	0.132	0.846	0.347
3sp	Chr7	157882037	Chr7_1	20	0.334	0.188	5158	1.02E-02	0.21	0.377	0.089	0.167	0.237	0.361	0.145	0.327	0.772	0.03	0.252	0.046	0.968	0.84	0.682	0.487	0.384	0.081	0.237	0.088	0.721	0.035	0.484	0.123
3sp	Chr7	153328122	Chr7_2	11	0.179	0.174	1587	4.00E-04	0.045	0	0.138	0.148	0.232	0.161	0.324	0.638	0.972	0.998	0.814	0.965	0.979	0.829	0.519	0.544	0.35	0.432	0.247	0.5	0.161	0	0.088	0
3sp	Chr7	153328122	Chr7_3	10	0.152	0.174	1990	4.00E-04	0.114	0	0.13	0.173	0.187	0.224	0.277	0.524	0.955	0.971	0.547	0.987	0.987	0.864	0.547	0.468	0.409	0.43	0.362	0.325	0.273	0	0.151	0.007
3sp	Chr1	3324448	Chr1_1	35	0.652	0.167	8341	0.0058	0.089	0.105	0.386	0.694	0.377	0.369	0.57	0.844	0.994	0.961	0.66	0.647	0.078	0	0.043	0	0.07	0.072	0.045	0.08	0.001	0	0.001	0
3sp	Chr9	208559564	Chr9_2	59	0.278	0.111	150251	1.40E-02	0.126	0.31	0.055	0.032	0.2	0.148	0.114	0.313	0.964	0.124	0.502	0.113	0.988	0.821	0.817	0.91	0.409	0.309	0.328	0.537	0.667	0.044	0.592	0.144
3sp	Chr20	366173761	Chr20_2	16	0.808	0.097	5684	9.00E-04	0.077	0.148	0.122	0.055	0.497	0.626	0.504	0.802	0.989	0.846	0.702	0.494	0.949	0.907	0.571	0.831	0.003	0	0.005	0.001	0.001	0	0.004	0
3sp	Chr3	63993066	Chr3_1	16	0.128	0.09	8367	2.10E-03	0.207	0.338	0	0.195	0.283	0.316	0.141	0.351	0.762	0.071	0.256	0.079	0.97	0.82	0.978	0.408	0.254	0.172	0.147	0.143	0.668	0.023	0.497	0.095
3sp	Chr18	353174923	Chr18_1	12	0.842	0.09	3383	4.10E-03	0.333	0.439	0	0.173	0.122	0.161	0.436	0.729	0.107	0.002	0.053	0.016	0.983	0.841	0.927	0.472	0.376	0.539	0.569	0.496	0.014	0	0.016	0
3sp	Chr4	96188022	Chr4_1	12	0.086	0.083	692	2.05E-02	0	0.148	0.409	0.529	0.283	0.307	0.377	0.691	0.993	0.868	0.946	0.489	0.049	0.001	0.031	0.008	0.236	0.184	0.149	0.157	0.05	0	0.042	0
3sp	Chr9	202542085	Chr9_1	55	0.527	0.056	469761	0.024	0.173	0.134	0.302	0.394	0.379	0.432	0.318	0.592	0.906	0.886	0.351	0.541	0.349	0.05	0.128	0.064	0.065	0.017	0.043	0.034	0.161	0	0.096	0.001
9sp	Chr20	451628138	Chr20_3	55	0.089	0.444	19197	1.00E-04	0.736	0.782	0.464	0.622	0.1	0.032	0.435	0.145	0	0	0	0	0.03	0	0.002	0	0.259	0.972	0.514	0.859	0.031	0.236	0.001	0.446
9sp	Chr4	109387633	Chr4_1	27	0.498	0.417	5519	1.00E-04	0.366	0.486	0.315	0.489	0.122	0.152	0.063	0.285	0.009	0	0.004	0.001	0.108	0.028	0.04	0.005	0.246	0.404	0.422	0.417	0.288	0.169	0.679	0.102
9sp	Chr18	395906216	Chr18_2	33	0.008	0.333	2856	1.00E-04	0.447	0.601	0.551	0.503	0.404	0.21	0.375	0.182	0	0	0	0	0	0.028	0	0.003	0.037	0.256	0.003	0.247	0.063	0.245	0.007	0.322
9sp	Chr20	436609240	Chr20_1	58	0.885	0.299	10410	4.00E-04	0.414	0.335	0.412	0.373	0.155	0.095	0.118	0.2	0	0.022	0.001	0.029	0.03	0.028	0.006	0.041	0.434	0.316	0.297	0.628	0.289	0.233	0.428	0.27
9sp	Chr18	408488264	Chr18_1	109	0.196	0.194	153576	8.00E-04	0.387	0.232	0.032	0	0.195	0.406	0.161	0.161	0.014	0.012	0.002	0.145	0.865	1	0.881	0.947	0.267	0.031	0.185	0.014	0.279	0.378	0.277	0.383
9sp	Chr14	311800447	Chr14_1	54	0.031	0.111	5783	1.00E-04	0.055	0.268	0.195	0.436	0.126	0.184	0.161	0.055	0.567	0.022	0.702	0.089	0.241	0.028	0.22	0.014	0.286	0.307	0.401	0.318	0.252	0.704	0.281	0.792
9sp	Chr13	288161750	Chr13_1	19	0.268	0.104	169638	1.36E-02	0.349	0.424	0.578	0.74	0.055	0	0.114	0.285	0.009	0	0.006	0.004	0	0	0	0	0.512	1	0.713	0.998	0.202	0.181	0.457	0.101
9sp	Chr3	80648276	Chr3_1	10	0.594	0.083	124	8.50E-03	0.369	0.399	0.212	0.31	0.13	0.11	0.263	0.095	0.009	0	0.003	0.007	0.279	0.121	0.182	0.099	0.402	0.285	0.377	0.568	0.137	0.368	0.067	0.628
9sp	Chr20	448139269	Chr20_2	14	0.572	0.083	104037	8.50E-03	0.385	0.391	0.114	0.184	0.197	0.182	0.321	0.045	0.014	0	0.002	0.009	0.433	0.13	0.48	0.358	0.267	0.313	0.176	0.325	0.092	0.738	0.023	0.845
9sp	Chr18	408488264	Chr18_1	109	0.196	0.15	153576	5.00E-04	0.387	0.232	0.032	0	0.195	0.406	0.161	0.161	0.016	0.009	0.002	0.132	0.852	1	0.879	0.945	0.269	0.025	0.181	0.011	0.314	0.237	0.272	0.366
9sp	Chr14	311800447	Chr14_2	54	0.031	0.133	5783	1.00E-04	0.055	0.268	0.195	0.436	0.126	0.184	0.161	0.055	0.549	0.026	0.699	0.079	0.237	0.033	0.216	0.011	0.277	0.339	0.396	0.301	0.246	0.687	0.276	0.784
9sp	Chr13	288161750	Chr13_1	19	0.268	0.111	169638	0.0146	0.349	0.424	0.578	0.74	0.055	0	0.114	0.285	0.008	<0.001	0.005	0.003	<0.001	<0.001	<0.001	<0.001	0.524	1	0.71	0.998	0.2	0.163	0.452	0.091
9sp	Chr3	80648276	Chr3_1	10	0.594	0.083	124	0.0091	0.369	0.399	0.212	0.31	0.13	0.11	0.263	0.095	0.008	<0.001	0.003	0.006	0.295	0.122	0.178	0.088	0.425	0.317	0.372	0.553	0.128	0.36	0.065	0.614
9sp	Chr20	448139269	Chr20_1	14	0.572	0.083	104037	0.0091	0.385	0.391	0.114	0.184	0.197	0.182	0.321	0.045	<0.001	<0.001	0.002	0.007	0.439	0.126	0.476	0.341	0.269	0.35	0.172	0.308	0.094	0.725	0.021	0.838

Sp: Species (3sp=three-spined sticlebacks and 9sp=nine-spined sticlebacks)

Chr: Chromosome

Region: matches outlier region in Figure 5 & 6, main text

Fst (Q95%) = 95% quantile among loci that mapped to the outlier regions from significant LD-clusters

Range=size of outlier genomic region in Bps

P_min=the lowest p-value among LD-clusters that mapped to this region

Subsequent column names should be interpreted as follows:

cor: correlation coefficient

P: P-value from EMMAX

A: relatedness was accounted for

unrl: individuals assumed unrelated

GC: genomic control was used to account for residual p-value inflation (otherwise permutation was used to assess significance)

Note that lineage was always included as co-factor for nine-spined sticklebacks

Supplementary Table 2 | Information on the nine-spined stickleback samples used in the study. The information on the three-spined stickleback samples is available at the Supplementary Table 1 in Fang et al. (2020).

Sample_ID	Population_ID	Ecotype	Region	GPS_N	GPS_E	Coverage
RUS-BOL-11	RUS-BOL	Freshwater	White and Barents Seas	66.3	33.4	17.59
RUS-BOL-12	RUS-BOL	Freshwater	White and Barents Seas	66.3	33.4	13.543
RUS-BOL-13	RUS-BOL	Freshwater	White and Barents Seas	66.3	33.4	15.704
RUS-BOL-6	RUS-BOL	Freshwater	White and Barents Seas	66.3	33.4	14.357
RUS-BOL-8	RUS-BOL	Freshwater	White and Barents Seas	66.3	33.4	15.359
RUS-BOL-9	RUS-BOL	Freshwater	White and Barents Seas	66.3	33.4	18.188
RUS-MAS-4	RUS-MAS	Freshwater	White and Barents Seas	66.3	33.4	10.793
RUS-MAS-5	RUS-MAS	Freshwater	White and Barents Seas	66.3	33.4	22.259
RUS-MAS-6	RUS-MAS	Freshwater	White and Barents Seas	66.3	33.4	11.077
RUS-MAS-7	RUS-MAS	Freshwater	White and Barents Seas	66.3	33.4	10.008
RUS-MAS-8	RUS-MAS	Freshwater	White and Barents Seas	66.3	33.4	10.732
RUS-MAS-9	RUS-MAS	Freshwater	White and Barents Seas	66.3	33.4	10.241
RUS-KRU-1	RUS-KRU	Freshwater	White and Barents Seas	66.3	33.4	10.448
RUS-KRU-11	RUS-KRU	Freshwater	White and Barents Seas	66.3	33.4	10.047
RUS-KRU-3	RUS-KRU	Freshwater	White and Barents Seas	66.3	33.4	10.287
RUS-KRU-4	RUS-KRU	Freshwater	White and Barents Seas	66.3	33.4	14.49
RUS-KRU-5	RUS-KRU	Freshwater	White and Barents Seas	66.3	33.4	34.787
RUS-KRU-9	RUS-KRU	Freshwater	White and Barents Seas	66.3	33.4	13.176
FIN-PUL-1	FIN-PUL	Freshwater	White and Barents Seas	69.967	27.967	16.138
FIN-PUL-2	FIN-PUL	Freshwater	White and Barents Seas	69.967	27.967	17.165
FIN-PUL-3	FIN-PUL	Freshwater	White and Barents Seas	69.967	27.967	18.294
FIN-PUL-4	FIN-PUL	Freshwater	White and Barents Seas	69.967	27.967	15.586
FIN-PUL-5	FIN-PUL	Freshwater	White and Barents Seas	69.967	27.967	14.841
FIN-PUL-6	FIN-PUL	Freshwater	White and Barents Seas	69.967	27.967	14.959
FIN-KEV-1	FIN-KEV	Freshwater	White and Barents Seas	69.75	27.017	14.231
FIN-KEV-2	FIN-KEV	Freshwater	White and Barents Seas	69.75	27.017	16.671
FIN-KEV-3	FIN-KEV	Freshwater	White and Barents Seas	69.75	27.017	17.047
FIN-KEV-4	FIN-KEV	Freshwater	White and Barents Seas	69.75	27.017	17.031
FIN-KEV-5	FIN-KEV	Freshwater	White and Barents Seas	69.75	27.017	14.689
FIN-KEV-8	FIN-KEV	Freshwater	White and Barents Seas	69.75	27.017	14.374
PP-VEN-AAN-2	VEN-AAN	Freshwater	White and Barents Seas	61.583	34.633	12.064
PP-VEN-AAN-8	VEN-AAN	Freshwater	White and Barents Seas	61.583	34.633	8.887
SWE-ABB-411	SWE-ABB	Freshwater	Baltic Sea	64.478	19.436	9.954
SWE-ABB-424	SWE-ABB	Freshwater	Baltic Sea	64.478	19.436	10.525
SWE-ABB-45	SWE-ABB	Freshwater	Baltic Sea	64.478	19.436	10.937
SWE-ABB-6	SWE-ABB	Freshwater	Baltic Sea	64.478	19.436	10.977
SWE-BYN-10	SWE-BYN	Freshwater	Baltic Sea	64.455	19.444	11.325
SWE-BYN-11	SWE-BYN	Freshwater	Baltic Sea	64.455	19.444	10.904
SWE-BYN-8	SWE-BYN	Freshwater	Baltic Sea	64.455	19.444	11.38
SWE-NAV-1	SWE-NAV	Freshwater	Baltic Sea	64.565	19.199	16.198
SWE-NAV-4	SWE-NAV	Freshwater	Baltic Sea	64.565	19.199	13.638
SWE-NAV-8	SWE-NAV	Freshwater	Baltic Sea	64.565	19.199	18.135
SWE-HAN-3	SWE-HAN	Freshwater	Baltic Sea	64.557	19.174	10.438
SWE-HAN-4	SWE-HAN	Freshwater	Baltic Sea	64.557	19.174	10.093
SWE-HAN-5	SWE-HAN	Freshwater	Baltic Sea	64.557	19.174	9.695
DEN-RES-1	DEN-RES	Freshwater	North Sea	56.183	9.633	12.722
DEN-RES-4	DEN-RES	Freshwater	North Sea	56.183	9.633	12.617
DEN-RES-6	DEN-RES	Freshwater	North Sea	56.183	9.633	12.551
PP-FIN-PAL-1	PP-FIN-PAL	Freshwater	White and Barents Seas	68.029	24.157	7.002
PP-FIN-PAL-2	PP-FIN-PAL	Freshwater	White and Barents Seas	68.029	24.157	6.851
PP-FIN-PAL-3	PP-FIN-PAL	Freshwater	White and Barents Seas	68.029	24.157	8.823
PP-FIN-PAL-4	PP-FIN-PAL	Freshwater	White and Barents Seas	68.029	24.157	4.618
PP-FIN-PAL-5	PP-FIN-PAL	Freshwater	White and Barents Seas	68.029	24.157	12.016
PP-FIN-PAL-6	PP-FIN-PAL	Freshwater	White and Barents Seas	68.029	24.157	16.961
PHY-NOR-STO-01	PP-NO-STO	Freshwater	Norwegian Sea	69.748	18.415	6.985
PHY-NOR-STO-02	PP-NO-STO	Freshwater	Norwegian Sea	69.748	18.415	7.84
PHY_NOR-STO-261	PP-NO-STO	Freshwater	Norwegian Sea	69.748	18.415	7.773
NOR-UGE-10	NOR-UGE	Freshwater	Norwegian Sea	63.958	10.427	16.52
NOR-UGE-11	NOR-UGE	Freshwater	Norwegian Sea	63.958	10.427	18.62
NOR-UGE-12	NOR-UGE	Freshwater	Norwegian Sea	63.958	10.427	17.442
NOR-UGE-13	NOR-UGE	Freshwater	Norwegian Sea	63.958	10.427	17.418
NOR-UGE-14	NOR-UGE	Freshwater	Norwegian Sea	63.958	10.427	17.632
NOR-UGE-15	NOR-UGE	Freshwater	Norwegian Sea	63.958	10.427	16.113
NOR-UGE-18	NOR-UGE	Freshwater	Norwegian Sea	63.958	10.427	16.045
NOR-UGE-1	NOR-UGE	Freshwater	Norwegian Sea	63.958	10.427	18.116
NOR-UGE-20	NOR-UGE	Freshwater	Norwegian Sea	63.958	10.427	17.078
NOR-UGE-21	NOR-UGE	Freshwater	Norwegian Sea	63.958	10.427	19.514
NOR-UGE-23	NOR-UGE	Freshwater	Norwegian Sea	63.958	10.427	20.286
NOR-UGE-24	NOR-UGE	Freshwater	Norwegian Sea	63.958	10.427	16.662
NOR-UGE-26	NOR-UGE	Freshwater	Norwegian Sea	63.958	10.427	17.674
NOR-UGE-27	NOR-UGE	Freshwater	Norwegian Sea	63.958	10.427	19.212
NOR-UGE-2	NOR-UGE	Freshwater	Norwegian Sea	63.958	10.427	16.919
NOR-UGE-5	NOR-UGE	Freshwater	Norwegian Sea	63.958	10.427	16.361
NOR-UGE-6	NOR-UGE	Freshwater	Norwegian Sea	63.958	10.427	16.295
NOR-UGE-7	NOR-UGE	Freshwater	Norwegian Sea	63.958	10.427	23.668
NOR-UGE-8	NOR-UGE	Freshwater	Norwegian Sea	63.958	10.427	20.279
NOR-UGE-9	NOR-UGE	Freshwater	Norwegian Sea	63.958	10.427	16.924
PHY-FRA-MAR-01	PP-FR-MAR	Freshwater	Western Europe	47.024	5.254	7.771
PHY-FRA-MAR-02	PP-FR-MAR	Freshwater	Western Europe	47.024	5.254	8.37
PP-NOR-AVE-2a	PP-NOR-AVE	Freshwater	North Sea	58.714	9.116	5.626
BEL-MAL-F26	BEL-MAL	Freshwater	Western Europe	51.175	3.469	12.671
BEL-MAL-F27	BEL-MAL	Freshwater	Western Europe	51.175	3.469	12.711
BEL-MAL-F28	BEL-MAL	Freshwater	Western Europe	51.175	3.469	11.554
BEL-MAL-F29	BEL-MAL	Freshwater	Western Europe	51.175	3.469	12.355
BEL-MAL-F30	BEL-MAL	Freshwater	Western Europe	51.175	3.469	12.33
PHY-SCO-HAR-01	PP-UK-HAR	Freshwater	Western Europe	55.754	-4.429	7.318
PHY-SCO-HAR-02	PP-UK-HAR	Freshwater	Western Europe	55.754	-4.429	8.785
PP-UK-LRE-1	PP-UK-LRE	Freshwater	Western Europe	57.611	-7.515	11.111
PP-UK-LRE-3	PP-UK-LRE	Freshwater	Western Europe	57.611	-7.515	11.053

PP-UK-LSC-1	PP-UK-LSC	Freshwater	Western Europe	57.584	-7.236	8.963
PP-UK-LSC-2	PP-UK-LSC	Freshwater	Western Europe	57.584	-7.236	6.467
GBR-GRO-1	GBR-GRO	Freshwater	Western Europe	57.615	-7.511	12.118
GBR-GRO-2	GBR-GRO	Freshwater	Western Europe	57.615	-7.511	12.232
GBR-GRO-3	GBR-GRO	Freshwater	Western Europe	57.615	-7.511	12.103
GBR-GRO-4	GBR-GRO	Freshwater	Western Europe	57.615	-7.511	12.2
GBR-GRO-5	GBR-GRO	Freshwater	Western Europe	57.615	-7.511	12.422
GBR-GRO-6	GBR-GRO	Freshwater	Western Europe	57.615	-7.511	12.163
PHY-CAN-TEM-01	PP-CA-TEM	Freshwater	Western Atlantic	47.714	-68.917	8.533
PHY-CAN-TEM-02	PP-CA-TEM	Freshwater	Western Atlantic	47.714	-68.917	10.296
PP-US-MAY-2	PP-US-MAY	Freshwater	Western Atlantic	43.343	-70.554	6.983
PP-US-MAY-3	PP-US-MAY	Freshwater	Western Atlantic	43.343	-70.554	6.87
PP-RU-MAG-1	PP-RU-MAG	Freshwater	Western Pacific	59.45	148.71	6.279
PP-RU-MAG-2	PP-RU-MAG	Freshwater	Western Pacific	59.45	148.71	8.457
PP-RU-KVA-1	PP-RU-KVA	Freshwater	Western Pacific	57.767	157.148	4.138
PP-RU-KVA-2	PP-RU-KVA	Freshwater	Western Pacific	57.767	157.148	4.785
PP-RU-KVA-3	PP-RU-KVA	Freshwater	Western Pacific	57.767	157.148	5.041
PP-RU-KVA-7	PP-RU-KVA	Freshwater	Western Pacific	57.767	157.148	8.152
PHY-RU-BLS-01	PP-RU-BLS	Freshwater	Western Pacific	52.757	156.253	7.69
PHY-RU-BLS-02	PP-RU-BLS	Freshwater	Western Pacific	52.757	156.253	6.968
PP-RU-GOR-6	PP-RU-GOR	Freshwater	Western Pacific	43.824	146.712	4.536
PP-RU-GOR-7	PP-RU-GOR	Freshwater	Western Pacific	43.824	146.712	5.324
PP-US-CRO-1	PP-US-CRO	Freshwater	Eastern Pacific	61.605	-149.511	11.364
PP-US-CRO-2	PP-US-CRO	Freshwater	Eastern Pacific	61.605	-149.511	8.74
USA-HLA-10	US-HLA	Freshwater	Eastern Pacific	61.591	-149.76	12.477
USA-HLA-11	US-HLA	Freshwater	Eastern Pacific	61.591	-149.76	12.482
USA-HLA-12	US-HLA	Freshwater	Eastern Pacific	61.591	-149.76	12.223
USA-HLA-13	US-HLA	Freshwater	Eastern Pacific	61.591	-149.76	12.286
USA-HLA-14	US-HLA	Freshwater	Eastern Pacific	61.591	-149.76	12.293
USA-HLA-15	US-HLA	Freshwater	Eastern Pacific	61.591	-149.76	12.541
USA-HLA-16	US-HLA	Freshwater	Eastern Pacific	61.591	-149.76	12.373
USA-HLA-17	US-HLA	Freshwater	Eastern Pacific	61.591	-149.76	12.389
USA-HLA-18	US-HLA	Freshwater	Eastern Pacific	61.591	-149.76	12.406
USA-HLA-19	US-HLA	Freshwater	Eastern Pacific	61.591	-149.76	12.418
USA-HLA-2	US-HLA	Freshwater	Eastern Pacific	61.591	-149.76	12.39
USA-HLA-20	US-HLA	Freshwater	Eastern Pacific	61.591	-149.76	12.535
USA-HLA-21	US-HLA	Freshwater	Eastern Pacific	61.591	-149.76	12.49
USA-HLA-3	US-HLA	Freshwater	Eastern Pacific	61.591	-149.76	12.432
USA-HLA-4	US-HLA	Freshwater	Eastern Pacific	61.591	-149.76	12.607
USA-HLA-5	US-HLA	Freshwater	Eastern Pacific	61.591	-149.76	12.624
USA-HLA-6	US-HLA	Freshwater	Eastern Pacific	61.591	-149.76	12.543
USA-HLA-7	US-HLA	Freshwater	Eastern Pacific	61.591	-149.76	12.51
USA-HLA-8	US-HLA	Freshwater	Eastern Pacific	61.591	-149.76	12.462
USA-HLA-9	US-HLA	Freshwater	Eastern Pacific	61.591	-149.76	12.433
GER-RUE-2	GER-RUE	Marine	Baltic Sea	54.402	13.21	13.705
GER-RUE-3	GER-RUE	Marine	Baltic Sea	54.402	13.21	11.102
GER-RUE-4	GER-RUE	Marine	Baltic Sea	54.402	13.21	11.011
GER-RUE-5	GER-RUE	Marine	Baltic Sea	54.402	13.21	23.822
GER-RUE-6	GER-RUE	Marine	Baltic Sea	54.402	13.21	23.714
GER-RUE-7	GER-RUE	Marine	Baltic Sea	54.402	13.21	15.042
1-f	FI-HEL	Marine	Baltic Sea	60.203	25.183	10.917
2-f	FI-HEL	Marine	Baltic Sea	60.203	25.183	11.228
3-f	FI-HEL	Marine	Baltic Sea	60.203	25.183	6.364
4-f	FI-HEL	Marine	Baltic Sea	60.203	25.183	6.284
5-f	FI-HEL	Marine	Baltic Sea	60.203	25.183	5.225
7-f	FI-HEL	Marine	Baltic Sea	60.203	25.183	10.015
SWE-BOL-60	SWE-BOL	Marine	Baltic Sea	63.661	20.212	9.355
SWE-BOL-61	SWE-BOL	Marine	Baltic Sea	63.661	20.212	11.618
SWE-BOL-62	SWE-BOL	Marine	Baltic Sea	63.661	20.212	10.719
SWE-BOL-63	SWE-BOL	Marine	Baltic Sea	63.661	20.212	11.58
SWE-BOL-64	SWE-BOL	Marine	Baltic Sea	63.661	20.212	12.116
SWE-BOL-65	SWE-BOL	Marine	Baltic Sea	63.661	20.212	11.034
FIN-KIV-1	FIN-KIV	Marine	Baltic Sea	65.008	25.436	16.484
FIN-KIV-2	FIN-KIV	Marine	Baltic Sea	65.008	25.436	15.889
FIN-KIV-3	FIN-KIV	Marine	Baltic Sea	65.008	25.436	15.52
FIN-KIV-5	FIN-KIV	Marine	Baltic Sea	65.008	25.436	14.902
FIN-KIV-6	FIN-KIV	Marine	Baltic Sea	65.008	25.436	16.813
FIN-KIV-7	FIN-KIV	Marine	Baltic Sea	65.008	25.436	17.262
RUS-LEV-10	RUS-LEV	Marine	White and Barents Seas	66.374	33.769	10.913
RUS-LEV-11	RUS-LEV	Marine	White and Barents Seas	66.374	33.769	10.919
RUS-LEV-3	RUS-LEV	Marine	White and Barents Seas	66.374	33.769	11.354
RUS-LEV-7	RUS-LEV	Marine	White and Barents Seas	66.374	33.769	11.347
RUS-LEV-8	RUS-LEV	Marine	White and Barents Seas	66.374	33.769	10.986
RUS-LEV-9	RUS-LEV	Marine	White and Barents Seas	66.374	33.769	11.37
DEN-NOR-10	DEN-NOR	Marine	North Sea	54.985	8.659	10.735
DEN-NOR-12	DEN-NOR	Marine	North Sea	54.985	8.659	12.122
DEN-NOR-13	DEN-NOR	Marine	North Sea	54.985	8.659	14.219
DEN-NOR-6	DEN-NOR	Marine	North Sea	54.985	8.659	11.014
DEN-NOR-7	DEN-NOR	Marine	North Sea	54.985	8.659	10.91
DEN-NOR-9	DEN-NOR	Marine	North Sea	54.985	8.659	11.192
DEN-NOR-14	DEN-NOR	Marine	North Sea	54.985	8.659	9.304
DEN-NOR-15	DEN-NOR	Marine	North Sea	54.985	8.659	9.52
DEN-NOR-16	DEN-NOR	Marine	North Sea	54.985	8.659	10.614
SWE-FIS-35	SWE-FIS	Marine	North Sea	58.233	11.4	12.396
SWE-FIS-36	SWE-FIS	Marine	North Sea	58.233	11.4	12.787
SWE-FIS-40	SWE-FIS	Marine	North Sea	58.233	11.4	12.669
SWE-FIS-42	SWE-FIS	Marine	North Sea	58.233	11.4	12.443
SWE-FIS-43	SWE-FIS	Marine	North Sea	58.233	11.4	18.584
SWE-FIS-45	SWE-FIS	Marine	North Sea	58.233	11.4	16.461
SWE-FIS-49	SWE-FIS	Marine	North Sea	58.233	11.4	15.865
SWE-FIS-52	SWE-FIS	Marine	North Sea	58.233	11.4	15.209
SWE-FIS-53	SWE-FIS	Marine	North Sea	58.233	11.4	12.614

Supplementary Table 3 | Populations in Figure 1b,c.

SPECIES	POP INDEX	POP ID	ECOTYPE	REGION	LOCATION
Three-spined stickeback	POP 1	Ran-M	Marine	Atlantic	EU
Three-spined stickeback	POP 2	BS-3sp	Marine	Atlantic	EU
Three-spined stickeback	POP 3	FIS	Marine	Atlantic	EU
Three-spined stickeback	POP 4	KRI	Marine	Atlantic	EU
Three-spined stickeback	POP 5	LEV	Marine	Atlantic	EU
Three-spined stickeback	POP 6	IND	Marine	Atlantic	EU
Three-spined stickeback	POP 7	SBJ	Marine	Atlantic	EU
Three-spined stickeback	POP 8	BAR	Marine	Atlantic	EU
Three-spined stickeback	POP 9	PRI	Marine	Atlantic	EU
Three-spined stickeback	POP 10	HAL	Marine	Atlantic	Western Atlantic
Three-spined stickeback	POP 11	FOR	Marine	Atlantic	Western Atlantic
Three-spined stickeback	POP 12	MAI	Marine	Atlantic	Western Atlantic
Three-spined stickeback	POP 13	SHI	Marine	Pacific	Western Pacific
Three-spined stickeback	POP 14	ASH	Marine	Pacific	Western Pacific
Three-spined stickeback	POP 15	KHA	Marine	Pacific	Western Pacific
Three-spined stickeback	POP 16	ANA	Marine	Pacific	Western Pacific
Three-spined stickeback	POP 17	BUT	Freshwater	Atlantic	EU
Three-spined stickeback	POP 18	QUI	Freshwater	Atlantic	EU
Three-spined stickeback	POP 19	KIN	Freshwater	Atlantic	EU
Three-spined stickeback	POP 20	MYR	Freshwater	Atlantic	EU
Three-spined stickeback	POP 21	MYV	Freshwater	Atlantic	EU
Three-spined stickeback	POP 22	FAR	Freshwater	Atlantic	EU
Three-spined stickeback	POP 23	TAK	Freshwater	Atlantic	EU
Three-spined stickeback	POP 24	SKF	Freshwater	Atlantic	EU
Three-spined stickeback	POP 25	NEV	Freshwater	Atlantic	EU
Three-spined stickeback	POP 26	SLI	Freshwater	Atlantic	EU
Three-spined stickeback	POP 27	VAT	Freshwater	Atlantic	EU
Three-spined stickeback	POP 28	ROT	Freshwater	Atlantic	EU
Three-spined stickeback	POP 29	KEV	Freshwater	Atlantic	EU
Three-spined stickeback	POP 30	KVA	Freshwater	Atlantic	EU
Three-spined stickeback	POP 31	MAS	Freshwater	Atlantic	EU
Three-spined stickeback	POP 32	BOL	Freshwater	Atlantic	EU
Three-spined stickeback	POP 33	LPD	Freshwater	Atlantic	Western Atlantic
Three-spined stickeback	POP 34	PF-3sp	Freshwater	Pacific	Western Pacific
Three-spined stickeback	POP 35	OTS	Freshwater	Pacific	Western Pacific
Three-spined stickeback	POP 36	OIR	Freshwater	Pacific	Western Pacific
Three-spined stickeback	POP 37	ALA	Freshwater	Pacific	Eastern Pacific
Three-spined stickeback	POP 38	MIS	Freshwater	Pacific	Eastern Pacific
Three-spined stickeback	POP 39	BEV	Freshwater	Pacific	Eastern Pacific
Three-spined stickeback	POP 40	PYE	Freshwater	Pacific	Eastern Pacific

SPECIES	POP INDEX	POP ID	ECOTYPE	REGION	LOCATION
Nine-spined stickeback	POP 1	SWE-FIS	Marine	Atlantic	EU
Nine-spined stickeback	POP 2	DEN-NOR	Marine	Atlantic	EU
Nine-spined stickeback	POP 3	RUS-LEV	Marine	Atlantic	EU
Nine-spined stickeback	POP 4	FIN-KIV	Marine	Atlantic	EU
Nine-spined stickeback	POP 5	SWE-BOL	Marine	Atlantic	EU
Nine-spined stickeback	POP 6	FI-HEL	Marine	Atlantic	EU
Nine-spined stickeback	POP 7	GER-RUE	Marine	Atlantic	EU
Nine-spined stickeback	POP 8	GBR-GRO	Freshwater	Atlantic	EU
Nine-spined stickeback	POP 9	PP-UK-LSC	Freshwater	Atlantic	EU
Nine-spined stickeback	POP 10	PP-UK-LRE	Freshwater	Atlantic	EU
Nine-spined stickeback	POP 11	PP-UK-HAR	Freshwater	Atlantic	EU
Nine-spined stickeback	POP 12	BEL-MAL	Freshwater	Atlantic	EU
Nine-spined stickeback	POP 13	PP-FR-MAR	Freshwater	Atlantic	EU
Nine-spined stickeback	POP 14	NOR-UGE	Freshwater	Atlantic	EU
Nine-spined stickeback	POP 15	PP-NO-STO	Freshwater	Atlantic	EU
Nine-spined stickeback	POP 16	PP-FIN-PAL	Freshwater	Atlantic	EU
Nine-spined stickeback	POP 17	DEN-RES	Freshwater	Atlantic	EU
Nine-spined stickeback	POP 18	SWE-HAN	Freshwater	Atlantic	EU
Nine-spined stickeback	POP 19	SWE-NAV	Freshwater	Atlantic	EU
Nine-spined stickeback	POP 20	SWE-BYN	Freshwater	Atlantic	EU
Nine-spined stickeback	POP 21	SWE-ABB	Freshwater	Atlantic	EU
Nine-spined stickeback	POP 22	VEN-AAN	Freshwater	Atlantic	EU
Nine-spined stickeback	POP 23	FIN-KEV	Freshwater	Atlantic	EU
Nine-spined stickeback	POP 24	FIN-PUL	Freshwater	Atlantic	EU
Nine-spined stickeback	POP 25	RUS-KRU	Freshwater	Atlantic	EU
Nine-spined stickeback	POP 26	RUS-MAS	Freshwater	Atlantic	EU
Nine-spined stickeback	POP 27	RUS-BOL	Freshwater	Atlantic	EU
Nine-spined stickeback	POP 28	PP-US-MAY	Freshwater	Atlantic	Western Atlantic
Nine-spined stickeback	POP 29	PP-CA-TEM	Freshwater	Atlantic	Western Atlantic
Nine-spined stickeback	POP 30	PP-RU-GOR	Freshwater	Pacific	Western Pacific
Nine-spined stickeback	POP 31	PP-RU-BLS	Freshwater	Pacific	Western Pacific
Nine-spined stickeback	POP 32	PP-RU-KVA	Freshwater	Pacific	Western Pacific
Nine-spined stickeback	POP 33	PP-RU-MAG	Freshwater	Pacific	Western Pacific
Nine-spined stickeback	POP 34	US-HLA	Freshwater	Pacific	Eastern Pacific
Nine-spined stickeback	POP 35	PP-US-CRO	Freshwater	Pacific	Eastern Pacific

SUPPLEMENTARY INFORMATION

Supplementary Information 1 | Methodological considerations

It is generally believed that strong genetic drift makes it difficult to detect signatures of parallel evolution (Galloway et al. 2020; Hoban et al. 2016; Matthey-Doret and Whitlock 2019) and therefore the reported differences between three- and nine-spined sticklebacks in parallel evolution could be argued to be an artefact of the inherent difficulty of detecting outlier loci among highly differentiated populations. However, both LDna-complexity reduction as well as using EMMAX to test for associations between SMLAs and ecotype are likely to mitigate the many shortcomings that otherwise characterise e.g. FST-based genome scan approaches (e.g. Bierne et al. 2011; Excoffier et al. 2009; Pritchard and Di Rienzo 2010). In our analyses, EMMAX produced test statistics that were highly correlated with both F_{ST} and CSS (we know already that CSS is correlated with F_{ST}; Jones et al. 2012), but importantly only when relatedness among individuals or other co-factors were not explicitly accounted for. *P*-value inflation in our analyses was high (up to λ~2), which was strongly reduced when relatedness was included as a random effect. However, both accounting for relatedness and correcting for p-value inflation (using GC) reduced the power to detect significant associations considerably, such that it was futile to find any outlier genomic regions without also using LDna-complexity reduction which allowed much fewer conservative corrections for multiple testing. Estimating and correcting for p-value inflation caused by relatedness is unfortunately not common practice in evolutionary genetic studies. However, nor is complexity reduction. It is thus possible that the increased power from p-value inflation roughly have counterbalanced the reduced power from unnecessarily conservative corrections for multiplicity in many previous studies. However, without re-analyses of these data sets, it is impossible to know to what extent previously detected outlier regions could in fact be artefacts of relatedness and p-value inflation.

We know that population structuring increases background differentiation in statistics estimating genetic differentiation between populations (Galloway et al. 2020; Hoban et al. 2016; Matthey-Doret and Whitlock 2019) and can also increase *p*-value inflation in genome wide association (GWA)-analyses when phenotypes are confounded by relatedness (Kang et al. 2010; Kang et al. 2008). There is thus reason to believe that these two observations are not independent, i.e. whenever background differentiation is high, we

can also expect p-value inflation due to relatedness. Importantly, however, accounting for relatedness can also increase power in GWA-studies (Kang et al. 2010; Kang et al. 2008). e.g. when multiple divergent populations segregate for the same causal variants for a given phenotype. This can also be expected when multiple populations in similar habitats display high frequencies of the same genetic variants (i.e. parallel evolution); the more divergent the populations are, the stronger will the contrast be between the neutral genetic background (reflecting relatedness) and the genomic regions under selection. For the outlier regions detected here, the overall effect sizes in three-spined sticklebacks differed when including relatedness as a random effect (0.38) compared to when relatedness was not accounted for (0.28) but remained roughly the same for nine-spined sticklebacks (0.29 *vs.* 0.27, respectively). Since p-value inflation (that reflect biases due to relatedness) in three-spined sticklebacks was higher (λ=1.95) compared to nine-spined sticklebacks (λ=1.73; when including lineage as co-factor), there is no reason to suspect that our methods lacked power to detect outlier regions in nine-spined sticklebacks across comparable levels of effect sizes in both species. Despite the fact that marine and freshwater individuals were pooled across all geographic regions in our analyses, no outlier region showed high effect sizes across all geographic regions. This demonstrates that, as long as effect sizes are sufficiently high overall, our methods also have sufficient power to detect regional parallelism (in both species). This is no different from detecting significant genotype-phenotype associations in GWA-studies for phenotypes that only segregate in only one or few of the populations included in the study.

While *p*-values are important, they tell very little about effect sizes which are much more likely to reflect the extent to which a given genomic region is affected by parallel evolution. While 26 and nine outlier genomic regions were significant in three-and nine-spined sticklebacks respectively, they were to different degrees sensitive to parameter settings and varied considerably in their effect sizes. These are all important metrics to consider when determining how significant a given genomic region harbouring freshwater adapted alleles are from an evolutionary/ecological perspective. However, while we can safely conclude that the marine-freshwater differentiated regions in three-spined sticklebacks outnumber such regions in nine-spined sticklebacks across a wide range of parameter/threshold values and correction methods, more detailed assessments of these outlier regions are outside the scope of this study.

Supplementary Information 2 | Outlier population (RUS-LEV) in the IBD analyses

The current marine pathway connecting White Sea and Baltic Sea through Barents and North Seas is unlikely to reflect the real geographic distance contributing to the close phylogenetic affinity between White Sea (RUS-LEV) and Baltic Sea populations sticklebacks in our data. This because the Baltic Sea populations are believed to be colonized from the White Sea after the last glaciation (see results, Fig. 3; Feng et al. 2020). In fact, the significant decrease in IBD when incorporating the White Sea RUS-LEV into the analysis (see below) is likely to be an artefact attributable to demographic history. In fact, the close relationship between the populations from the Baltic Sea and White Sea was likely a result of the southward colonisation during the retreat of the Scandinavian ice sheet in the late Pleistocene and early Holocene (Feng et al. 2020; Glückert 1995; Guo et al. 2019). Based on this assumption, the results of IBD analyses were discussed based on the dataset excluding the outlier population RUS-LEV.

When incorporating White Sea population (RUS-LEV) of the nine-spined stickleback into the IBD analyses, the IBD of marine nine-spined sticklebacks decreased by a factor of 3.1 (MLPE, β = 1.2e-4 *vs*. 4.0e-5; *Supplementary Information Fig. 1*). However, the IBD of nine-spined sticklebacks was still 7.7 times stronger than that of three-spined sticklebacks in marine habitat (MLPE, β = 4.0e-5 *vs.* 5.1e-6; *Supplementary Information Fig. 1*).

Supplementary Information Figure 1 | Isolation-by-distance (IBD) in three- and nine-spined stickleback populations. This dataset includes the nine-spined stickleback White Sea population (RUS-LEV). IBD was tested across (a) marine and (b) freshwater three- (red) and nine-spined stickleback (blue) populations using maximum-likelihood population-effects (MLPE) model. The results of the regression coefficient (β) suggest stronger IBD in nine- than in three-spined sticklebacks: 7.7 times in the marine habitat and 23.6 times in the freshwater habitat (see results). IBD comparisons were restricted to European populations (see methods).

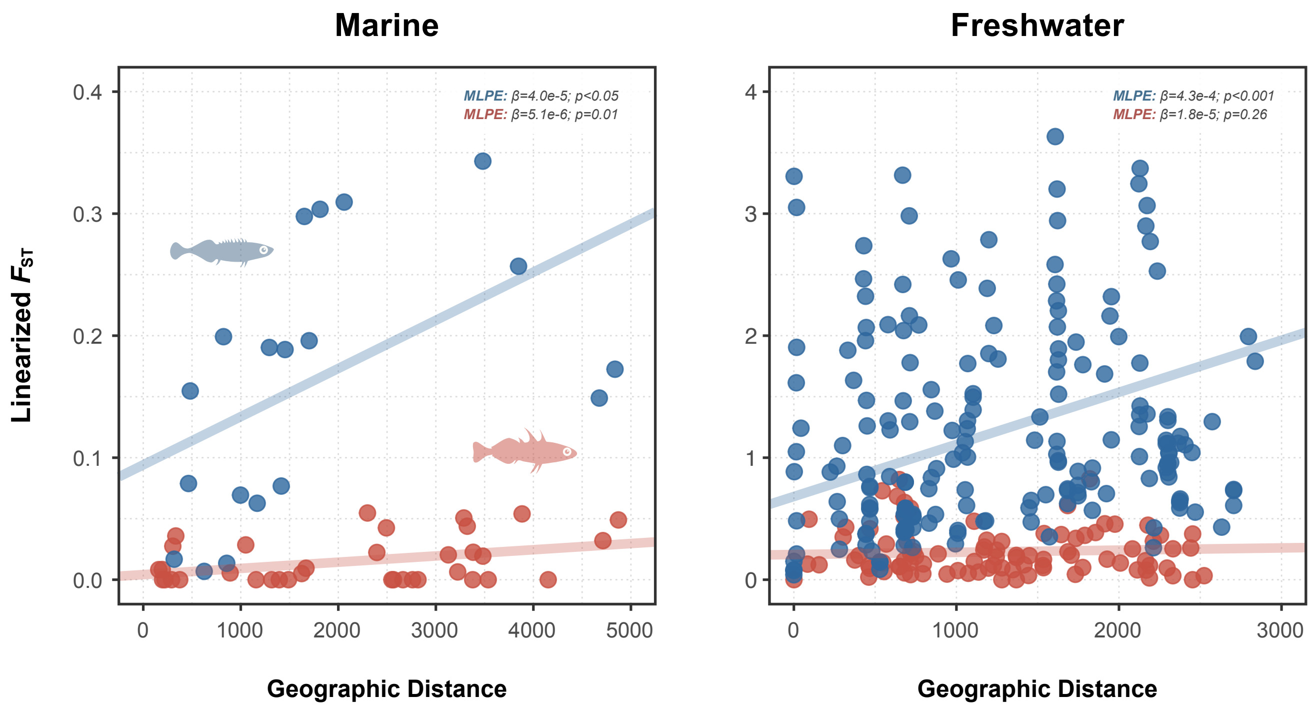
Marine
Freshwater
Linearized F_{ST}
MLPE: β=4.0e-5; p<0.05
MLPE: β=5.1e-6; p=0.01
MLPE: β=4.3e-4; p<0.001
MLPE: β=1.8e-5; p=0.26
0.4
0.3
0.2
0.1
0.0
0
1000
2000
3000
4000
5000
4
3
2
1
0
0
1000
2000
3000
Geographic Distance
Geographic Distance

References in the Supplementary Materials

Bierne N, Welch J, Loire E, Bonhomme F, David P. 2011. The coupling hypothesis: why genome scans may fail to map local adaptation genes. *Mol Ecol*. 20:2044-2072.

Bouckaert RR. 2010. DensiTree: making sense of sets of phylogenetic trees. *Bioinformatics*. 26:1372-1373.

Charlesworth B, Morgan M, Charlesworth D. 1993. The effect of deleterious mutations on neutral molecular variation. *Genetics*. 134:1289-1303.

Cruickshank TE, Hahn MW. 2014. Reanalysis suggests that genomic islands of speciation are due to reduced diversity, not reduced gene flow. *Mol Ecol*. 23:3133-3157.

Danecek P et al. 2011. The variant call format and VCFtools. *Bioinformatics*. 27:2156-2158.

Drummond AJ, Suchard MA, Xie D, Rambaut A. 2012. Bayesian phylogenetics with BEAUti and the BEAST 1.7. *Mol Biol Evol*. 29:1969-1973.

Dutoit L, Vijay N, Mugal CF, Bossu CM, Burri R, Wolf J, Ellegren H. 2017. Covariation in levels of nucleotide diversity in homologous regions of the avian genome long after completion of lineage sorting. *Proc Royal Soc B*. 284:20162756.

Efron B. 2010. Correlated z-values and the accuracy of large-scale statistical estimates. *J Am Stat Assoc*. 105:1042-1055.

Excoffier L, Hofer T, Foll M. 2009. Detecting loci under selection in a hierarchically structured population. *Heredity (Edinb)*. 103:285-298.

Fang B, Kemppainen P, Momigliano P, Feng X, Merila J. 2020. On the causes of geographically heterogeneous parallel evolution in sticklebacks. *Nat Ecol Evol*. 4:1105-1115.

Feng X, Merilä J, Löytynoja A. 2020. We shall meet again - Genomics of historical admixture in the sea. *bioRxiv*.

Galloway J, Cresko WA, Ralph P. 2020. A few stickleback suffice for the transport of alleles to new lakes. *G3: Genes, Genomes, Genetics*. 10:505-514.

Glückert G. 1995. The Baltic Ice Lake in south Finland and its outlets. *Quaternary international*. 27:47-51.

Goudet J. 2005. Hierfstat, a package for R to compute and test hierarchical F-statistics. *Mol Ecol Notes*. 5:184-186.

Guo B, Fang B, Shikano T, Momigliano P, Wang C, Kravchenko A, Merilä J. 2019. A phylogenomic perspective on diversity, hybridization and evolutionary affinities in the stickleback genus Pungitius. *Mol Ecol*. 28:4046-4064.

Hoban S et al. 2016. Finding the Genomic Basis of Local Adaptation: Pitfalls, Practical Solutions, and Future Directions. *Am Nat*. 188:379-397.

Jones FC et al. 2012. The genomic basis of adaptive evolution in threespine sticklebacks. *Nature*. 484:55-61.

Joo JWJ, Hormozdiari F, Han B, Eskin E. 2016. Multiple testing correction in linear mixed models. *Genome Biol*. 17:1-18.

Kang HM et al. 2010. Variance component model to account for sample structure in genome-wide association studies. *Nat Genet*. 42:348-354.

Kang HM, Zaitlen NA, Wade CM, Kirby A, Heckerman D, Daly MJ, Eskin E. 2008. Efficient control of population structure in model organism association mapping. *Genetics*. 178:1709-1723.

Kemppainen P et al. 2015. Linkage disequilibrium network analysis (LDna) gives a global view of chromosomal inversions, local adaptation and geographic structure. *Mol Ecol Resour*. 15:1031-1045.

Kemppainen P et al. 2017. Controlling for P-value inflation in allele frequency change in experimental evolution and artificial selection experiments. *Mol Ecol Resour*. 17:770-782.

Kingman GAR et al. 2020. Predicting future from past: The genomic basis of recurrent and rapid stickleback evolution. *bioRxiv*.

Kitano J et al. 2009. A role for a neo-sex chromosome in stickleback speciation. *Nature*. 461:1079.

Korneliussen TS, Albrechtsen A, Nielsen R. 2014. ANGSD: Analysis of next generation sequencing data. *BMC Bioinform*. 15:356.

Li Z, Kemppainen P, Rastas P, Merilä J. 2018. Linkage disequilibrium clustering-based approach for association mapping with tightly linked genomewide data. *Mol Ecol Resour*. 18:809-824.

Matthey-Doret R, Whitlock MC. 2019. Background selection and FST: consequences for detecting local adaptation. *Mol Ecol*. 28:3902-3914.

Momigliano P, Florin AB, Merila J. 2021. Biases in demographic modelling affect our understanding of recent divergence. *Mol Biol Evol*. In press.

Natri HM, Merila J, Shikano T. 2019. The evolution of sex determination associated with a chromosomal inversion. *Nat Commun*. 10:145.

Natri HM, Shikano T, Merilä J. 2013. Progressive recombination suppression and differentiation in recently evolved neo-sex chromosomes. *Mol Biol Evol*. 30:1131-1144.

Nei M. 1987. Molecular evolutionary genetics. *Columbia university press*.

Nei M, Li WH. 1979. Mathematical model for studying genetic variation in terms of restriction endonucleases. *Proc Natl Acad Sci U S A*. 76:5269-5273.

Nelson TC, Cresko WA. 2018. Ancient genomic variation underlies repeated ecological adaptation in young stickleback populations. *Evol Lett*. 2:9-21.

Pembleton LW, Cogan NOI, Forster JW. 2013. StAMPP: an R package for calculation of genetic differentiation and structure of mixed-ploidy level populations. *Mol Ecol Resour*. 13:946-952.

Pritchard JK, Di Rienzo A. 2010. Adaptation - not by sweeps alone. *Nat Rev Genet*. 11:665-667.

Rastas P, Calboli FCF, Guo B, Shikano T, Merilä J. 2015. Construction of ultradense linkage maps with Lep-MAP2: stickleback F 2 recombinant crosses as an example. *Genome Biol Evol*. 8:78-93.

Ravinet M et al. 2017. Interpreting the genomic landscape of speciation: a road map for finding barriers to gene flow. *J Evol Biol*. 30:1450-1477.

Robinson MR, Wray NR, Visscher PM. 2014. Explaining additional genetic variation in complex traits. *Trends Genet*. 30:124-132.

Santure AW, Garant D. 2018. Wild GWAS—association mapping in natural populations. *Mol Ecol Resour*. 18:729-738.

Savolainen O, Lascoux M, Merilä J. 2013. Ecological genomics of local adaptation. *Nat Rev Genet*. 14:807-820.

Shapiro MD et al. 2009. The genetic architecture of skeletal convergence and sex determination in ninespine sticklebacks. *Curr Biol*. 19:1140-1145.

Vinkhuyzen AA, Wray NR, Yang J, Goddard ME, Visscher PM. 2013. Estimation and partition of heritability in human populations using whole-genome analysis methods. *Annu Rev Genet*. 47:75-95.
